# Naming Potentially Endangered Parasites: Foliicolous Mycobiota of *Dimorphandra wilsonii*, a Highly Threatened Brazilian Tree Species

**DOI:** 10.1371/journal.pone.0147895

**Published:** 2016-02-24

**Authors:** Meiriele da Silva, Danilo B. Pinho, Olinto L. Pereira, Fernando M. Fernandes, Robert W. Barreto

**Affiliations:** 1 Departamento de Fitopatologia, Universidade Federal de Viçosa, Viçosa, Minas Gerais, Brazil; 2 Fundação Zoo-Botânica de Belo Horizonte, Belo Horizonte, Minas Gerais, Brazil; The University of Wisconsin - Madison, UNITED STATES

## Abstract

A survey of foliicolous fungi associated with *Dimorphandra wilsonii* and *Dimorphandra mollis* (Fabaceae) was conducted in the state of Minas Gerais, Brazil. *Dimorphandra wilsonii* is a tree species native to the Brazilian Cerrado that is listed as critically endangered. Fungi strictly depending on this plant species may be on the verge of co-extinction. Here, results of the pioneering description of this mycobiota are provided to contribute to the neglected field of microfungi conservation. The mycobiota of *D*. *mollis*, which is a common species with a broad geographical distribution that co-occurs with *D*. *wilsonii*, was examined simultaneously to exclude fungal species occurring on both species from further consideration for conservation because microfungi associated with *D*. *wilsonii* should not be regarded as under threat of co-extinction. Fourteen ascomycete fungal species were collected, identified, described and illustrated namely: *Byssogene wilsoniae* sp. nov., *Geastrumia polystigmatis*, *Janetia dimorphandra-mollis* sp. nov., *Janetia wilsoniae* sp. nov., *Johansonia chapadiensis*, *Microcalliopsis dipterygis*, *Phillipsiella atra*, *Piricauda paraguayensis*, *Pseudocercospora dimorphandrae* sp. nov., *Pseudocercosporella dimorphandrae* sp. nov., *Ramichloridiopsis wilsoniae* sp. and gen. nov., *Stomiopeltis suttoniae*, *Trichomatomyces byrsonimae* and *Vesiculohyphomyces cerradensis*. Three fungi were exclusively found on *D*. *wilsonii* and were regarded as potentially threatened of extinction: *B*. *wilsoniae*, *J*. *wilsoniae* and *R*. *wilsoniae*.

## Introduction

The Cerrado is a savannah-like Brazilian biome that is second in area only to the Amazon forest. It covers 21% of the country (2 million km^2^) and is largely coincident with the central plateau [1]. However, it is rapidly being replaced by crops, pastures and exotic forest plantations [2, 3]. One of the many endemic plant species occurring in this biome that are now endangered is *Dimorphandra wilsonii* Rizzini (Fabaceae), which is a tree known as “faveiro de Wilson”. Only12 individuals of this tree species were known in nature, all in highly disturbed sites in privately owned areas and most in two neighboring municipalities in the Brazilian state of Minas Gerais (Paraopeba and Caetanópolis) [4] *Dimorphandra wilsonii* is listed in the IUCN Red List (http://www.iucnredlist.org/) as being critically endangered, which is the highest level of risk for the survival of a species prior to extinction in nature.

The general lack of public knowledge and awareness about fungi and their significance and the cryptic nature of most fungi, which are either invisible to the naked eye or produce ephemeral macroscopic fruit bodies, has probably led to the mistaken impression that species belonging to the kingdom Fungi are capable of escaping environmental changes because they are easily dispersed, ubiquitous and broadly spread [5]. Nevertheless, surveys of environmental DNA have indicated that microbial communities have a well-defined structure, with populations that have a high level of endemism [6]. However, the practical difficulties for gathering evidence that individual fungal species are actually threatened and a lack of effort by scientists in general (including mycologists) have resulted in the virtual absence of fungi from the lists of endangered species and from policies aimed at preventing global loss of biodiversity. Minter [7] referred to fungi as “the orphans of Rio” because these organisms were left out of the agenda of the Earth Summit (United Nations Conference on Environment and Development—UNCED) that occurred in Rio de Janeiro in June, 1992. Some years ago, Rocha et al. [8] conjectured that it might be possible to gather convincing scientific evidence of the status of “threatened of extinction” for certain members of the kingdom Fungi by investigating highly host-specific plant pathogenic fungi associated with endangered plant species. The loss of one rare plant species may lead to coextinction events that threaten a range of specialized organisms that depend strictly on that species for their survival. Such events are well documented for parasite-host interactions, such as pigeon lice, primate parasites, pollinizer wasps and herbivorous insects [9, 10, 11], but not for fungi.

It was only recently that the need for conservation of fungi was embraced as a duty by mycologists. The first international conference on the issue took place in Whitby, UK, in October, 2009 (“Fungal conservation science, infrastructure and politics”). The International Society for Fungal Conservation was founded (http://www.fungal-conservation.org/) on August 6, 2010, during the 9th International Mycological Congress. More recently, the Third International Congress on Fungal Conservation took place in Gökova Bay, Turkey, in 2013 (http://www.fungal-conservation.org/icfc3).

The first report addressing the issue of fungal conservation in Brazil was published by Rocha et al. [8] and involved the study of the foliage mycobiota of *Coussapoa flocosa* Akkermans & C.C. (Cecropiaceae). The mycobiota was regarded by the authors as likely to be in danger of coextinction due to its dependence on this rare endemic tree in the Brazilian Atlantic Forest. This study led to the discovery of six new fungal species, including a new fungal genus.

The present work aims to expand the study begun by Rocha et al. [8] to encompass an additional endangered Brazilian plant species (*Dimorphandra wilsonii*) and its mycobiota, which may also be potentially endangered by coextinction. Additionally, we also studied the mycobiota of *Dimorphandra mollis* Benth., which is a common species with a broad geographical distribution that is closely related to *D*. *wilsonii* and coexists with that plant in its remaining area of occurrence in nature. This study was performed to determine which fungi occurring on *D*. *wilsonii* also occurred on the non-endangered *D*. *mollis*. Such species should not deserve further consideration for conservation because the microfungi associated with *D*. *wilsonii* should not be regarded as being in danger of co-extinction.

Hence, the objectives of this study were: I) to survey and describe the foliicolous mycobiota associated with *Dimorphandra wilsonii*; II) to survey and describe the mycobiota of *D*. *mollis*; III) to verify the possibility of the co-occurrence of fungi on *D*. *wilsonii* and *D*. *mollis* and; IV) to produce a preliminary list of fungal species on *D*. *wilsonii* that are potentially in danger of extinction based on this evidence.

## Materials and Methods

Survey trips were conducted between 2009 and 2011 in the municipalities of Paraopeba, Caetanópolis, Juatuba, Fortuna de Minas, Sete Lagoas and Pequi (Table 1).

**Table 1:** Collecting sites for *D*. *wilsonii* and *D*. *mollis*.

Localities	Dates	Coordenates in degrees-min-seg
Paraopeba- Fazenda Tabuleiro Grande	13 Jul 2009; 19 Jul 2010; 21 Jul 2010; 27 Jul 2011;	44°W 25' 57"–19°S 15' 24"; 44°W 25' 55,6"–19° S15' 21,4"
Paraopeba-Flona Paraopeba	15 Jul 2009; 20–21 Jul 2010	44°W 24' 18.41146''; 19°S 15' 43.15176''
Caetanópolis-Fazenda São Bento	15 Jul 2009; 27 Jul 2011	44°W 25' 27"–19°S 19' 51"; 44°W 25' 25.2503''–19°S 19' 48''
Juatuba—Fazenda Coqueiros	07 Feb 2011;	44°W 24' 42"–19°S 56' 52"; 44°W 24' 40.3882''–19°S 56' 50''
Fortuna de Minas—Sitio Grota D’agua	07 Feb 2011;	44°W 27' 55"–19°S 33' 28"; 44°W 27' 57.3010''–19°S 33' 27''; 44°W 27' 58.0934'–19°S 33' 28''
Sete Lagoas—Faz. Sta Terezinha	09 Feb 2011;	44°W 12' 18"–19°S 31' 10"; 44°W 12' 24"–19°S 31' 00"; 44°W 12' 18.9638''–19°S 31' 10''; 44°W 12' 25.3354''; 19°S 31' 06''
Pequi—Fazenda Areião	08 Dez 2011	44°W 35' 56.1779''; 19°S 33' 00.5779''
Pequi—Fazenda Alvorada	08 Dez 2011	44°W 35' 37.4828''; 19°S 34' 08''

**Fig 1:**
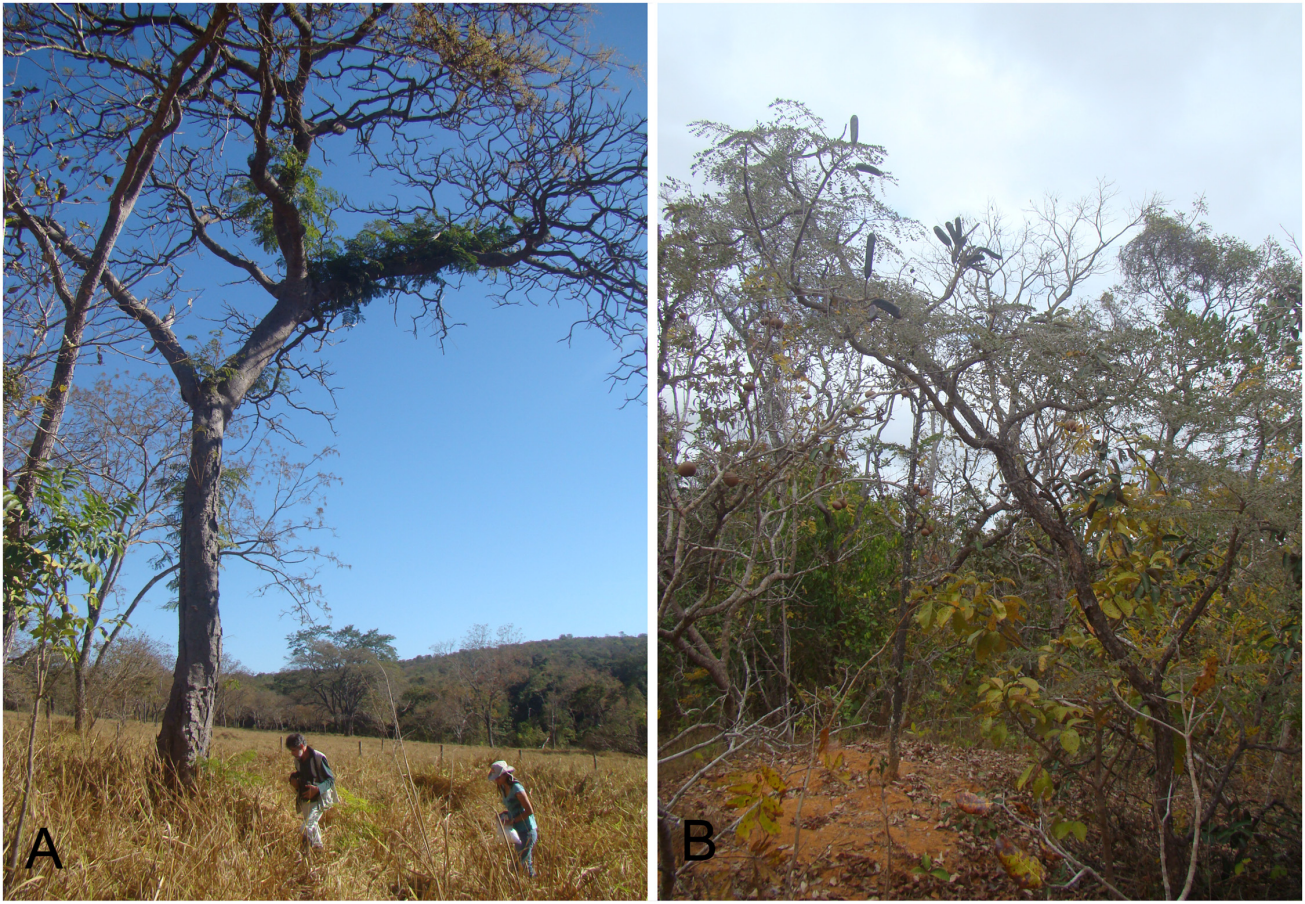
Individuals of the two plant species surveyed during the work: *Dimorphandra wilsonii*–critically endangered species—in a disturbed situation (exotic grass pasture) (A) and *Dimorphandra mollis*–common undangered species—in a protected fragment of Brazilian Cerrado (B).

The owners of each property allowed samples to be collected for study, and no special permits were required for this study other than the registration of the corresponding author in SISBIO—ICMBio, Ministerio do Meio Ambiente (Reg. number 1839773). The fungi collected during this study have no official status of endangered or protected species at this stage at any level. Existing information on the localities of the occurrence of *D*. *wilsonii* individuals in nature were provided by F. Fernandes based on his regular surveys for remaining individuals of this species that started in 2003 [12]. Whenever individuals belonging to the closely related species *D*. *mollis* were found growing in the vicinities of an individual of *D*. *wilsonii*, branches and foliage of individuals belonging to that species were also collected. *Dimorphandra wilsonii* is readily separated from *D*. *mollis* by having longer pods with a sweetish scent and paler gray bark that is not easily detached unlike *D*. *mollis*. Its leaflets are also larger as compared with *D*. *mollis* (3–5 cm long) (Fig 1). Pictures were taken in the field with a SONY DSC-H9 digital camera, and samples of branches bearing foliage were collected with a long-poled pruner and dried in a plant press.

After screening in the lab, dried relevant specimens were deposited at the herbarium of Universidade Federal de Viçosa (VIC). Samples were examined under a stereomicroscope (OLYMPUS SZX7) while still fresh, and fungal structures were either scraped with a scalpel from tissue surfaces or free-hand sections of fungal structures or sections prepared using a freezing microtome (Microm HM 520) were mounted in lactophenol or other mounting media. Observations,measurements and drawing were performed with an OLYMPUS BX 51 light microscope fitted with a digital camera (OLYMPUS E330) and a drawing tube.

Isolations of fungi in pure cultures were attempted by the direct transfer of spores or other fungal structures onto plates containing VBA (vegetable broth agar) as described by Pereira et al. [13] with the help of a sterile fine-point needle. Pure cultures were preserved in PCA slants or silica-gel as described by Dhingra and Sinclair [14] and were deposited in the culture collection of the Universidade Federal de Viçosa (COAD).

For scanning electron microscopy, samples were placed in a critical point dryer (Baltec model 030) with CO_2_ as the transition fluid; after drying, the samples were coated with gold (20 nm thick) with a sputter coater (Balzers^®^ model FDU 010) and examined with a Carl-Zeiss Model LEO VP 1430 electron microscope.

Culture descriptions were based on observations of the colonies formed in plates containing potato dextrose agar (PDA) or potato carrot agar (PCA). The plates were incubated at 25°C under a 12 h daily light regimen (light provided by two white and one near-UV lamps placed 35 cm above the plates) for 23 days. The color terminology followed Rayner [15].

For the molecular phylogeny studies of selected fungi, pure cultures were grown on PDA at 25°C for up to four weeks depending on their growth rate. Genomic DNA was extracted from the mycelium using the Wizard^®^ Genomic DNA Purification Kit (Promega Corporation, WI, USA) following the manufacturer’s instructions. For the sequencing of *Phillipsiella atra*, DNA was extracted by removing fungal structures from the plant tissue with a fine glass needle and placing them in a microtube (1.5 ml) provided by the extraction kit. Each fungal structure was carefully examined under the highest power of a stereomicroscope to check for possible contamination with other fungi or mycoparasites and to exclude any plant material from the sample. PCR reactions included the following ingredients for each 25 μl reaction: 12.5 μl of 2X DreamTaq^™^ PCR Master Mix (MBI Fermentas, Vilnius, Lithuania), 1 μl of 10 μM of each forward and reverse primer synthesized by Invitrogen (Carlsbad, USA), a maximum of 25 ng/μl of genomic DNA, and nuclease-free water to complete the total volume.

The primers ITS4 (5’- TCCTCCGCTTATTGATATGC -3’) and ITS5 (5’- GGAAGTAAAAGTCGTAACAAGG -3’) were used to amplify the internal transcribed spacer region (ITS) of the nuclear ribosomal RNA operon, including the 3’ end of the 18S rRNA, the first internal transcribed spacer region, the 5.8S rRNA gene, the second internal transcribed spacer region and the 5’ end of the 28S rRNA gene [16]. The large ribosomal subunit (LSU) was amplified with the primer pair LR0R (5’-ACCCGCTGAACTTAAGC-3’) and LR5 (5’-TCCTGAGGGAAACTTCG -3’) [17]. The amplifications were performed with a BIO RAD C1000 (Thermal Cycler) with an initial denaturation at 95°C for 5 min, followed by 40 cycles of denaturation at 94°C for 1 min, annealing at 60°C for ITS and 53°C for LSU for 45 s, extension at 72°C for 2 min and a final extension of 7 min at 72°C. The amplified products were visualized on a 1% agarose gel to check the product size and purity. PCR products were purified with the PEQLAB E.Z.N.A.^®^ Cycle-Pure Kit following the manufacturer’s protocol. The sequencing was performed directly from the purified PCR-amplified fragments using the automated sequencer MegaBACE 500TM. The nucleotide sequences were edited with the BioEdit software [18]. All sequences were checked manually, and nucleotides with ambiguous positions were clarified using primers targeting both sequence directions. New sequences were deposited in GenBank (http://www.ncbi.nlm.nih.gov). The large ribosomal subunit sequences of additional species were retrieved from GenBank (Table 2).

**Table 2:** GenBank acession numbers of LSU rDNA sequences derived from strains used in the phylogenetic analysis.

Spicies	Isolate	Genbank acession n° LSU	References
*Pseudocercospora eucalyptorum*	CMW5228	DQ204762	Hunter et al. 2006
*Pseudocercospora robusta*	CMW5151	DQ204767	Hunter et al. 2006
*Mycosphaerella gracilis*	CBS243.94	DQ204750	Hunter et al. 2006
*Mycosphaerella bixae*	CBS111804	GU214455	Crous et al. 2009
*Mycosphaerella bixae*	CPC2554	GU214455	Crous et al. 2009
*Pseudocercospora natalensis*	CBS111069	DQ267576	Hunter et al. 2006
*Pseudocercospora palleobrunnea*	CBS124771	GQ303319	Cheewangkoon et al. 2009
*Pseudocercospora leucadendri*	CPC1869	GU214480	Crous et al. 2009
*Pseudocercospora crousii*	CBS119487	GU253729	Crous et al. 2013
*Pseudocercospora cymbidiicola*	CBS115132	GU253733	Crous et al. 2013
*Pseudocercospora ixorae*	CBS118760	GU253759	Crous et al. 2013
*Pseudocercospora lyoniae*	MUCC910	GU253768	Crous et al. 2013
*Pseudocercospora metrosideri*	CBS118795	GU253774	Crous et al. 2013
*Pseudocercospora myrticola*	MUCC632	GU253777	Crous et al. 2013
*Pseudocercospora fori*	CPC14880	GU253824	Crous et al. 2013
*Pseudocercospora proteae*	CPC15217	GU253826	Crous et al. 2013
*Pseudocercospora theae*	CBS128.30	GU253838	Crous et al. 2013
*Pseudocercospora libertiae*	CBS114643	JQ324959	Crous et al. 2013
*Pseudocercospora subulata*	CBS118489	JX901907	Quaedvlieg et al. 2012
*Mycosphaerella fori*	CMW9095	DQ204748	Hunter et al. 2006
*Pseudocercospora basitruncata*	CBS 111280	DQ204760	Hunter et al. 2006
*Pseudocercospora arecacearum*	CBS118792	GU253703	Crous et al. 2013
*Pseudocercospora coprosmae*	CBS114639	JQ324946	Crous et al. 2013
*Mycosphaerella sphaerulinae*	CPC4314	GU214451	Crous et al. 2009
*Pseudocercospora sphaerulinae*	CBS112621	JX901906	Quaedvlieg et al. 2012
*Pseudocercospora cruenta*	CBS462.75	GU214473	Crous et al. 2009
*Pseudocercospora vitis*	CPC11595	JX901912	Quaedvlieg et al. 2012
*Mycosphaerella graminicola*	CBS110744	EU019298	Crous et al. 2007
*Mycosphaerella punctiformis*	CBS113265	AY490776	Verkley et al. 2004
*Mycosphaerella ellipsoidea*	CMW5166	DQ246254	Hunter et al. 2006
Mycosphaerella endophytica	CBS111519	DQ246255	Hunter et al. 2006
Mycosphaerella stromatosa	CBS101953	EU167598	Simon et al. 2009
*Mycosphaerella gregaria*	CBS110501	DQ246251	Hunter et al. 2006
*Mycosphaerella wachendorfiae*	CPC18338	JF951163	Crous et al. 2011
*Paramycosphaerella brachystegia*	CPC21136	KF777230	Crous et al. 2013
*Mycosphaerella rosigena*	CBS330.51	GU214413	Crous et al. 2009
*Mycosphaerella marksii*	CPC13724	GQ852615	Crous et al. 2009
*Mycosphaerella marksii*	CMW5230	DQ246246	Hunter et al. 2006
*Mycosphaerella intermedia*	CMW7164	DQ246248	Hunter et al. 2006
*Pseudocercospora epispermogonia*	CBS110750	DQ204757	Hunter et al. 2006
*Zasmidium cellare*	CBS146.36	EU041878	Arzanlou et al. 2007
*Zasmidium nocoxi*	CPC14044	GQ852735	Crous et al. 2009
*Zasmidium xenoparkii*	CBS111185	JF700966	Quaedvlieg et al. 2012
*Mycosphaerella aleuritidis*	CBS282.62	EU167594	Simon et al. 2009
*Ramichloridium cerophilum*	CBS103.59	EU041855	Arzanlou et al. 2007
*Mycosphaerella vietnamensis*	AGI099A	EU882134	Cheewangkoon et al. 2008
*Mycosphaerella vietnamensis*	CBS119974	JF700944	Quaedvlieg et al. 2012
*Mycosphaerella parkii*	CBS387.92	AY152619	Verkley et al. 2004
*Schizothyrium pomi*	CBS406.61	EF134949	Batzer et al. 2008
*Schizothyrium pomi*	CBS486.50	EF134948	Batzer et al. 2008
*Schizothyrium pomi*	CBS228.57	EF134947	Batzer et al. 2008
*Johansonia chapadiensis*	CBS128068	HQ423450	Crous et al. 2010
*Radulidium subulatum*	CBS287.84	EU041844	Arzanlou et al. 2007
*Radulidium epichloes*	CBS361.63	EU041842	Arzanlou et al. 2007
*Ramichloridium epichloes*	MUCL3124	AF050287	Untereiner & Naveau 1999
*Pseudovirgaria hyperparasitica*	CPC10704	EU041823	Arzanlou et al. 2007
*Pseudovirgaria hyperparasitica*	CPC10702	EU041822	Arzanlou et al. 2007
*Pseudovirgaria grisea*	CPC19126	JF957610	Braun et al. 2011
*Pseudovirgaria grisea*	CPC19130	JF957612	Braun et al. 2011
*Dothidea sambuci*	AFTOL-ID 274	AY544681	Schoch et al. 2009
***Phillipsiella atra***	**VIC 31773**	**KJ459711**	**present work**
***Piricauda paraguayensis***	**VIC 31782**	**KJ459712**	**present work**
***Pseudoramichloridium wilsoniae***	**VIC 31803**	**KJ459713**	**present work**
***Janetia wilsoniae***	**VIC 31772**	**KJ459714**	**present work**
***Janetia wilsoniae***	**VIC 31780**	**KJ459715**	**present work**
***Pseudocercospora dimorphandrae***	**VIC 31774**	**KJ459716**	**present work**
***Pseudocercospora dimorphandrae***	**VIC 31775**	**KJ459717**	**present work**
***Pseudocercosporella dimorphandrae***	**VIC 31788**	**KJ459718**	**present work**
***Pseudocercosporella dimorphandrae***	**VIC 31776**	**KJ459719**	**present work**

Consensus regions were compared against GenBank’s database using their Mega BLAST program. The closest sequence hits were downloaded in FASTA format and aligned using the multiple sequence alignment program MUSCLE^®^ [19] with default parameters. MUSCLE^®^ was implemented using the MEGA v.5 software [20]. The alignments were checked, and manual adjustments were made where necessary.

**Fig 2:**
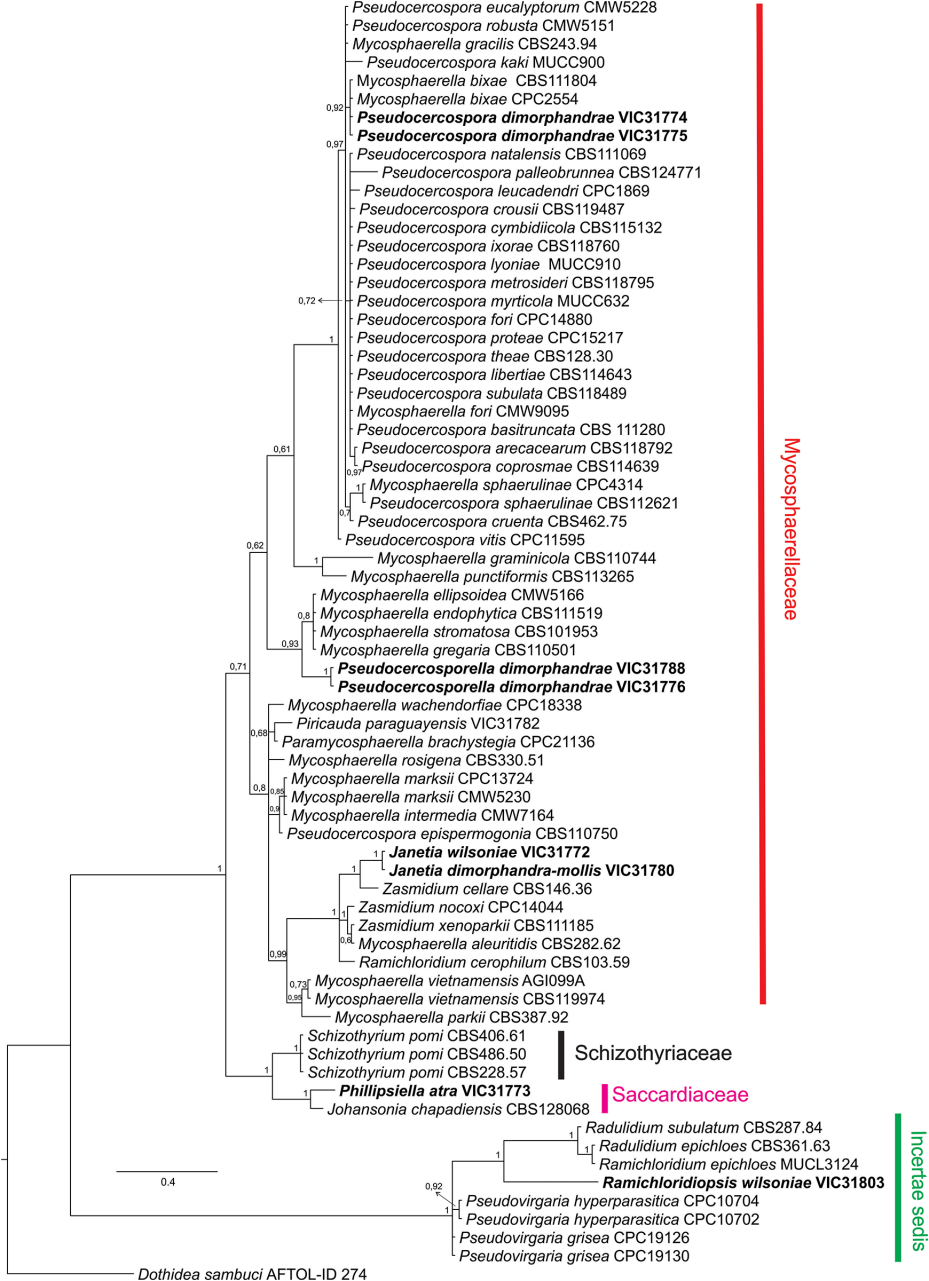
Hypothesis about the phylogenetic placement of selected fungi among those collected on *Dimorphandra wilsonii* and *Dimorphandra mollis* and their connection with related fungi derived from Bayesian analysis of partial nuclear large subunit ribosomal DNA gene sequences. Bayesian posterior probabilities are indicated at the nodes. The tree was rooted with *Dothidea sambuci* AFTOL-ID274. Isolates with newly obtained sequences for this study are given in bold.

Bayesian inference (BI) analyses employing a Markov Chain Monte Carlo method were performed. MrMODELTEST 2.3 [21] was used to select the nucleotide substitution models for the BI analysis. We used the general time-reversible model of evolution [22], including the estimation of invariable sites and assuming a discrete gamma distribution with six rate categories (GTR+I+G). The BI analysis was performed with MrBayes v. 3.1.1 [23, 24, 25, 26]. Four MCMC (Markov chain Monte Carlo) chains were run simultaneously starting from random trees for 10,000,000 generations. Trees were sampled every 1000th generation for a total of 10,000 trees. The first 2500 trees were discarded as the burn-in phase of each analysis. Posterior probabilities [23] were determined from a majority-rule consensus tree generated with the remaining 7500 trees. Convergence of the log likelihoods was analyzed with TRACER v. 1.4.1 (beast.bio.ed.ac.uk/Tracer); no indication of a lack of convergence was detected. This analysis was repeated three times starting from different random trees to ensure trees from the same tree space were sampled during each analysis. Trees were visualized in FigTree (http://tree.bio.ed.ac.uk/software/figtree/) and exported to graphics programs. The tree was rooted to *Dothidea sambuci* (Pers.) Fr. (AFTOL-ID274) (Fig 2).

### Nomenclature

The electronic version of this article in Portable Document Format (PDF) in a work with an ISSN or ISBN will represent a published work according to the International Code of Nomenclature for algae, fungi, and plants, and hence the new names contained in the electronic publication of a PLOS ONE article are effectively published under that Code from the electronic edition alone, so there is no longer any need to provide printed copies. In addition, new names proposed in this work were submitted to MycoBank from where they will be made available to the Global Names Index. The unique MycoBank number can be resolved and the associated information viewed through any standard web browser by appending the MycoBank number contained in this publication to the prefix http://www.mycobank.org/MB/. The online version of this work is archived and available from the following digital repositories: [PubMed Central, LOCKSS etc].

## Results and Discussion

### Phylogeny

Amplification of the partial LSU was selected for the molecular phylogenetic identification of the new species included in this study. The manually adjusted alignment included 70 taxa and contained 768 characters, of which 195 were parsimony-informative, 230 were variable and 534 were conserved. Although the ITS sequences were not used in the phylogenetic analyses, they were deposited in GenBank for future studies and DNA barcode purposes (Table 2).

### Taxonomy

Fourteen fungal species were found to be associated with *D*. *wilsonii*, and six species were collected in association with *D*. *mollis*. These fungi are described below.

**Fig 3:**
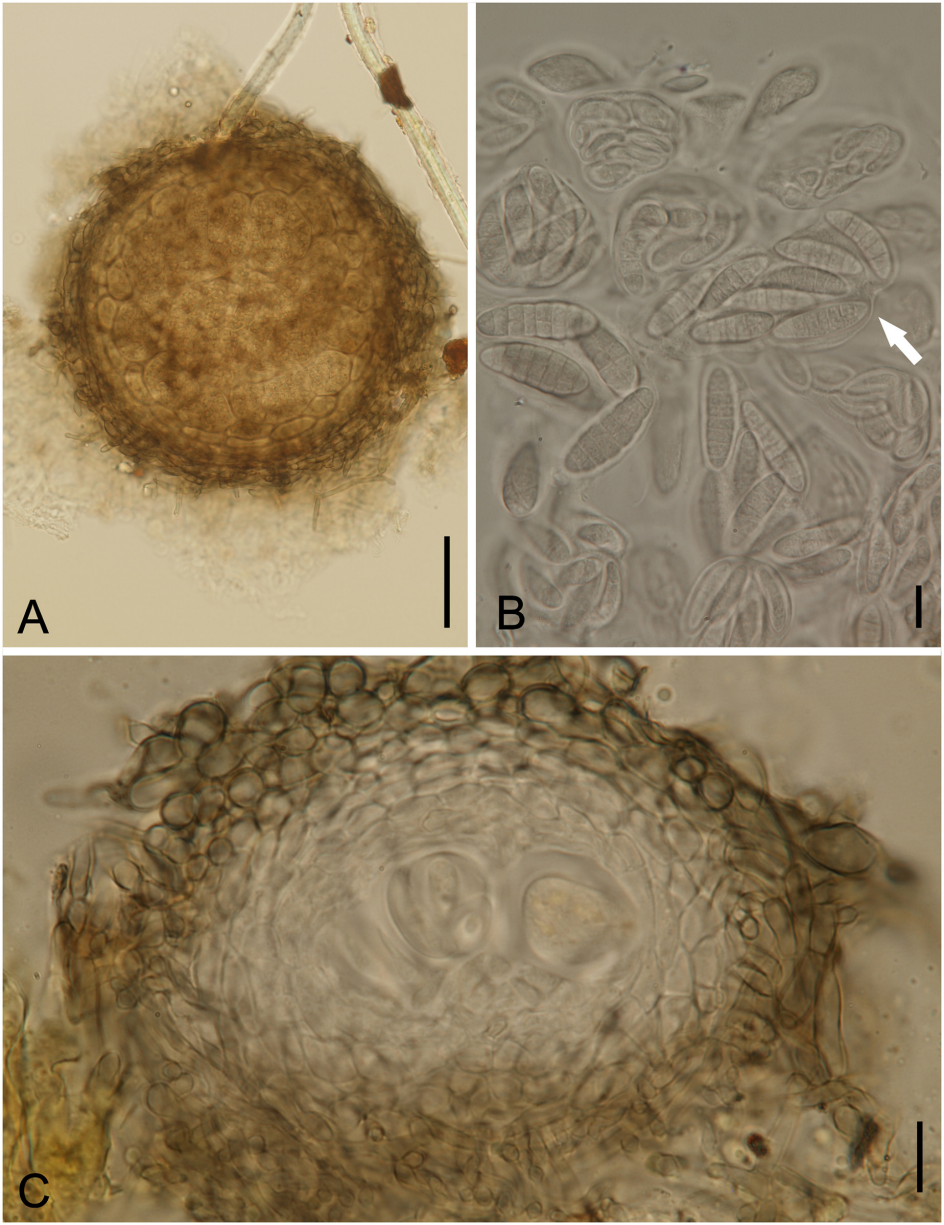
*Byssogene wilsoniae* on *Dimorphandra wilsonii*. (A) Discoid, non ostiolate ascoma surrounded by a net-forming external mycelium. (B) Bitunicate, 8-spored asci with cylindrical to ellipsoid, dictyoseptate, subhyaline ascospores—arrowed. (C) Cross section of ascoma showing asci. Bars: 50 μm (A); 10 μm (B); 20 μm (C).

*Byssogene wilsoniae* M. Silva & R.W. Barreto, sp.nov. Fig 3

[urn:lsid:mycobank.org:names: 810520]

*Etymology*. In reference to the specific epithet of the host *Dimorphandra wilsonii*

Colonies on living leaves, hypophyllous, sparse, not aggregated, causing no disease symptoms. Internal mycelium indistinct. External mycelium hypogenous, anastomosing, net-forming, slightly undulate, composed of pale brown, flattened, thin walled, septate hyphae, 2.5–5.0 μm, smooth. Ascomata pseudothecia hyophyllous, superficial, solitary, discoid, 175–225 μm diam., 100–117.5 μm high, non-ostiolate, black, margin raised, outer wall of textura globulosa gray becoming hyaline, inner wall pseudoparenchymatose, 6-cell thick, 12.5–20 μm, dark brown. Asci bitunicate, ovate, oblong to obclavate, 37.5–65 × 25–35 μm, 8–spored. Ascospores cylindrical to ellipsoid, 20–27.5×5–10 μm, dictyoseptate, subhyaline, guttulate, smooth.

*Specimens examined*: On living leaves of *Dimorphandra wilsonii*. BRAZIL: Minas Gerais: Paraopeba, Fazenda Tabuleiro Grande, 27 Jul 2011, M. Silva (VIC 31808 − HOLOTYPE).

*Notes*: The fungus described above is a member of the family Saccardiaceae and bears discoid superficial ascomata, parallel bitunicate asci in a single layer, and ascospores with two or many cells that are hyaline or brown, which is typical for the family [26]. Among the genera in Saccardiaceae, this fungus clearly fits into the genus *Byssogene* Syd. because it has a combination of dictyoseptate, almost hyaline ascospores, discoid ascomata and superficial brown mycelium [27]. *Byssogene amboinensis* Syd. was the only species in this genus and is known to occur on *Eugenia* sp. (Myrtaceae) in the Ambon Islands, Indonesia [28]. *Byssogene amboinensis* differs from our species by having shorter and narrower asci (40–52 × 18–25 μm) and shorter ascospores (15–17 μm). Therefore, our fungus clearly represents a new species for this genus. The fungus was only found on *D*. *wilsonii* at a single location on a single plant.

*Geastrumia polystigmatis* Bat. & M.L.Farr, *Saccardoa* 1: 71 (1960) Fig 4

**Fig 4:**
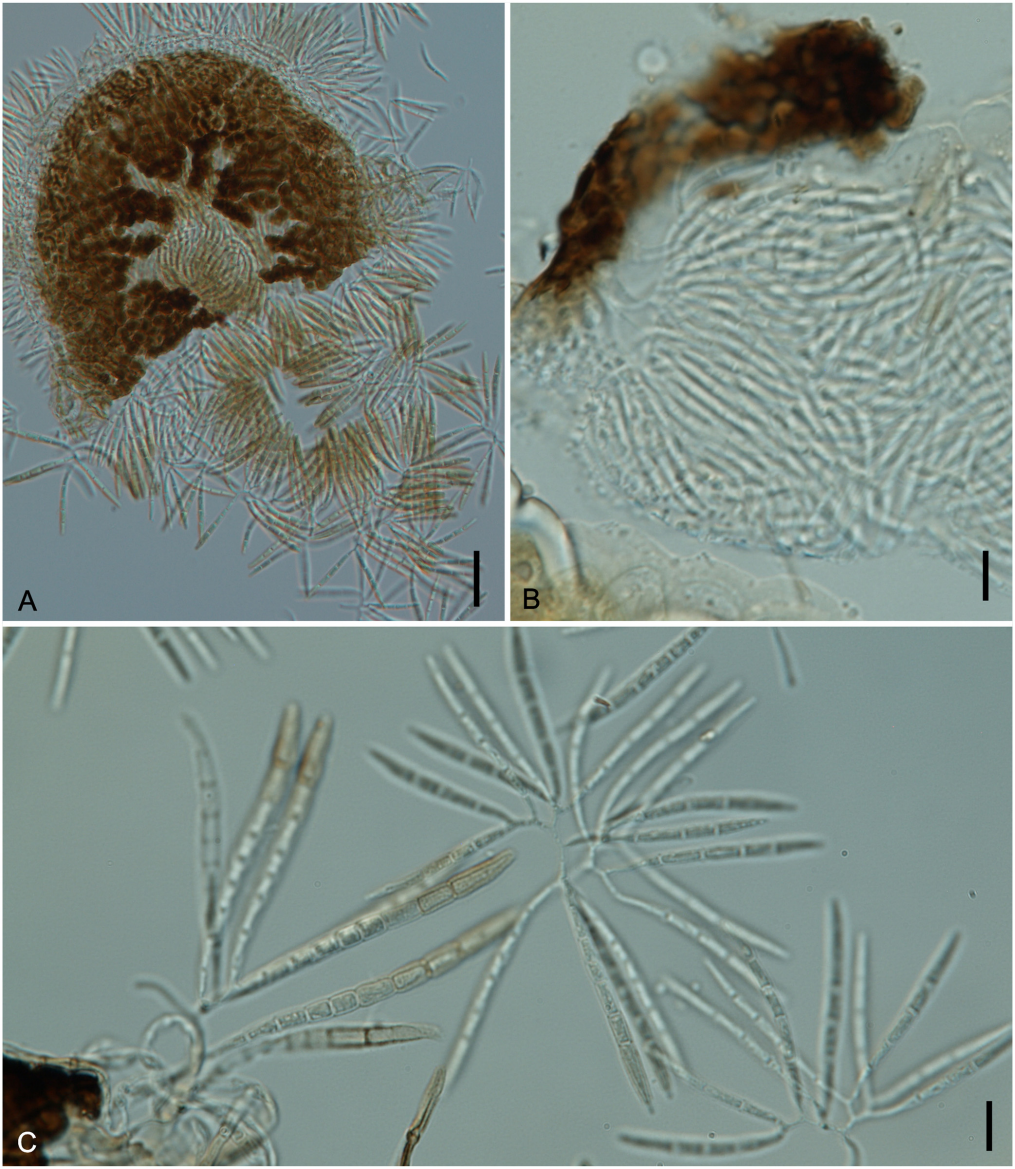
*Geastrumia polystigmatis* on *Dimorphandra wilsonii*. (A) Conidioma opening by irregular rupture of wall, releasing groups of conidia. (B) Cross section of conidioma showing conidiogenous cells developing from the hyphae into the upper wall—arrowed. (C) Cheiroid conidia with fasciculate group of filiform arms attached to a common, branched basal stalk cell. Bars: 20 μm (A); 10 μm (B, C).

Colonies on living leaves, adaxial, circular to irregular, sparse. Internal mycelium indistinct. External mycelium loose, branched, 2.5–3 μm diam, discrete and irregularly scattered over areas of the host, septate, medium brown to sub-hyaline, smooth. Conidiomata hypophyllous, superficial, hemispherical or subglobose, 125–140 μm diam., 52.5–85 μm in height, scutelate, opening by an irregular rupture, wall composed of one-celled layer of textura angularis, 10–12.5 μm, dark brown, smooth. Conidiophores micronematous, conidiogenous cells, holoblastic, narrowly elongate ampulliform, clavate to fusiform, 8–17 × 2 μm, swelling at the apex to produce a more or less dichotomous forking and a cluster of clavate to fusiform outgrowths which differentiates into conidial arms after the initial cluster has been cut off from the conidiogenous cell by a septum, hyaline, smooth. Conidia dry, holoblastic, solitary, cheiroid, fasciculate in groups of 4–10 straight to slightly curved filiform arms closely united by a long pedicellate basal cell, each arm, 15–50 × 2–4 μm, apex 2.5–4 μm, base 1–2.5 μm, 2–7 septate, hyaline when immature becoming light brown at maturity, smooth.

*Specimens examined*: On living leaves of *Dimorphandra wilsonii*. BRAZIL: Minas Gerais: Paraopeba, Fazenda Tabuleiro Grande, 13 Jul 2009, M. Silva (VIC 31771).

*Notes*: *Geastrumia polyastigmatis* Bat. & M.L. Farr is the only species in this genus. This species was previously known to occur on a member of the Fabaceae—*Andira jamaicensis* (W. Wright) Urb. in Brazil and the Dominican Republic, as well as on other unrelated host species, including *Costus afer* Ker Gawl. (Costaceae) and *Hymenocardia acida* Tul. (Phyllantaceae) in Tanzania [29]. The fungus on *D*. *wilsonii* fits well within the description of *G*. *polyastigmatis* and is reported here for the first time on this host.

**Fig 5:**
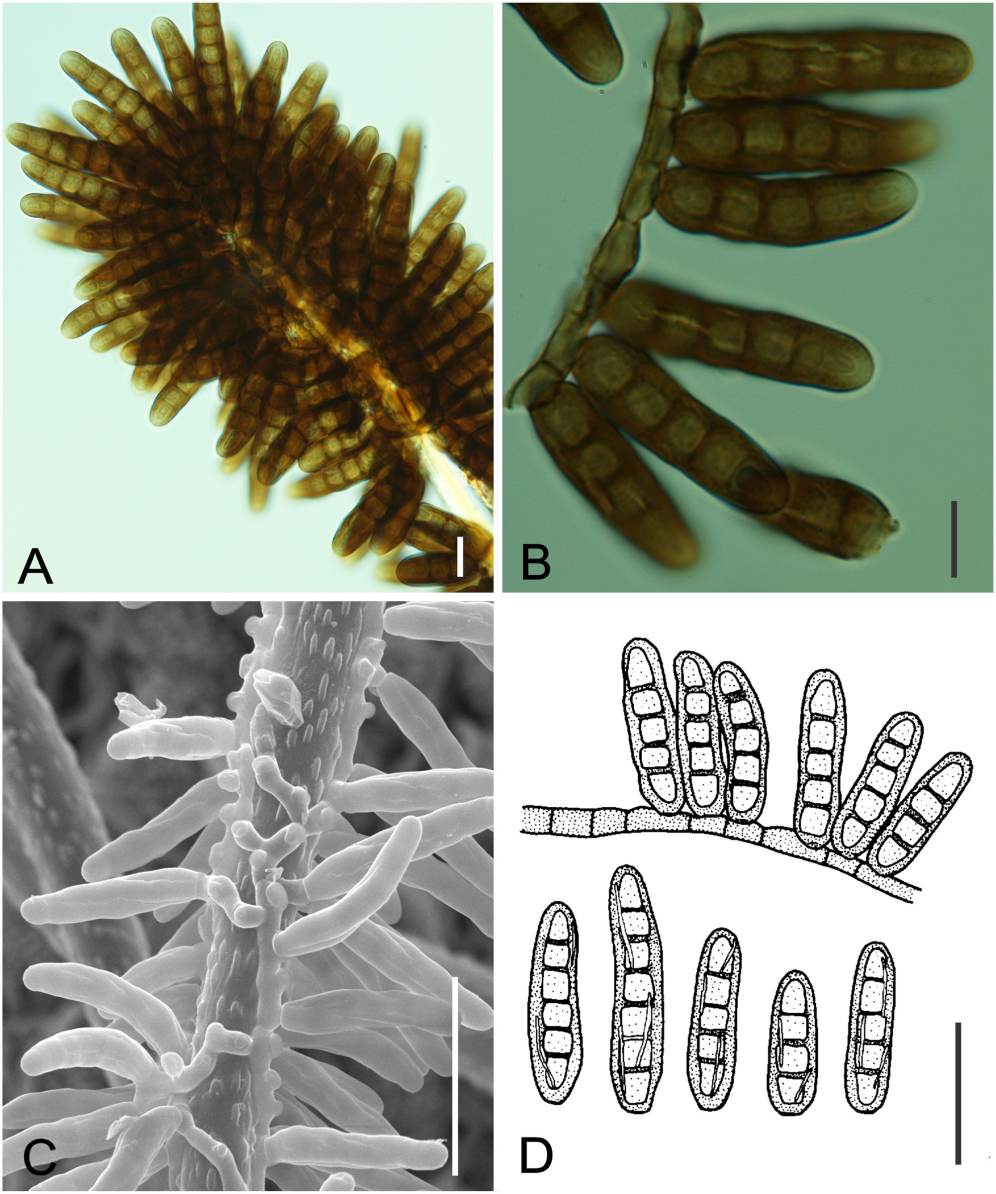
*Janetia dimorphandra-mollis* on *Dimorphandra mollis*. (A) Dense coloniy on upper portion of trichome. (B) Cylindrical distoseptate conidia attached to conidiogeous cells. (C) SEM of colony showing external mycelium with humped micronematous conidiogenous cells and conidia. (D) External hyphae with row of conidiogenous cells bearing conidia and some loose conidia. Bars: 10 μm (A, C, D); 5 μm (B).

*Janetia dimorphanda-mollis* M. Silva & R.W. Barreto, sp. nov. Fig 5

[urn:lsid:mycobank.org:names: 810523]

*Etymology*. In reference to the host species *Dimorphandra mollis*

Colonies hypophyllous, on trichomes, effuse, brown or dark brown, producing a bottle brush appearance (as observed in microscope mounts). Internal mycelium indistinct. Superficial mycelium growing on trichomes and foliar surface, 3–5 μm diam, branched, septate, brown, smooth. Conidiophores micronematous, restricted to fertile nearly undifferentiated humped hyphal cells, 5–6 × 3–4 μm, brown, smooth. Conidiogenous cells holoblastic, monoblastic, subdenticulate. Conidiogenous loci a flat-topped hump on conidiogenous cells, 1.5–3 μm, unthickened, dark brown. Conidia dry, holoblastic, solitary, cylindrical, straight to slightly curved, often slightly constricted at septae, 16–35 × 6.5–9.5 μm, apex rounded 4–5 μm, base 4.5–6 μm, 2–7 transversally distoseptate, hilum inconspicuous, eguttulate, pale brown to brown, smooth walled but faintly spirally sulcate along the conidial length.

*Specimens examined*: On living leaves of *Dimorphandra mollis*. BRAZIL: Minas Gerais: Paraopeba, Floresta Nacional de Paraopeba, 21 Jul 2010, M. Silva (VIC 31812 − HOLOTYPE).

**Fig 6:**
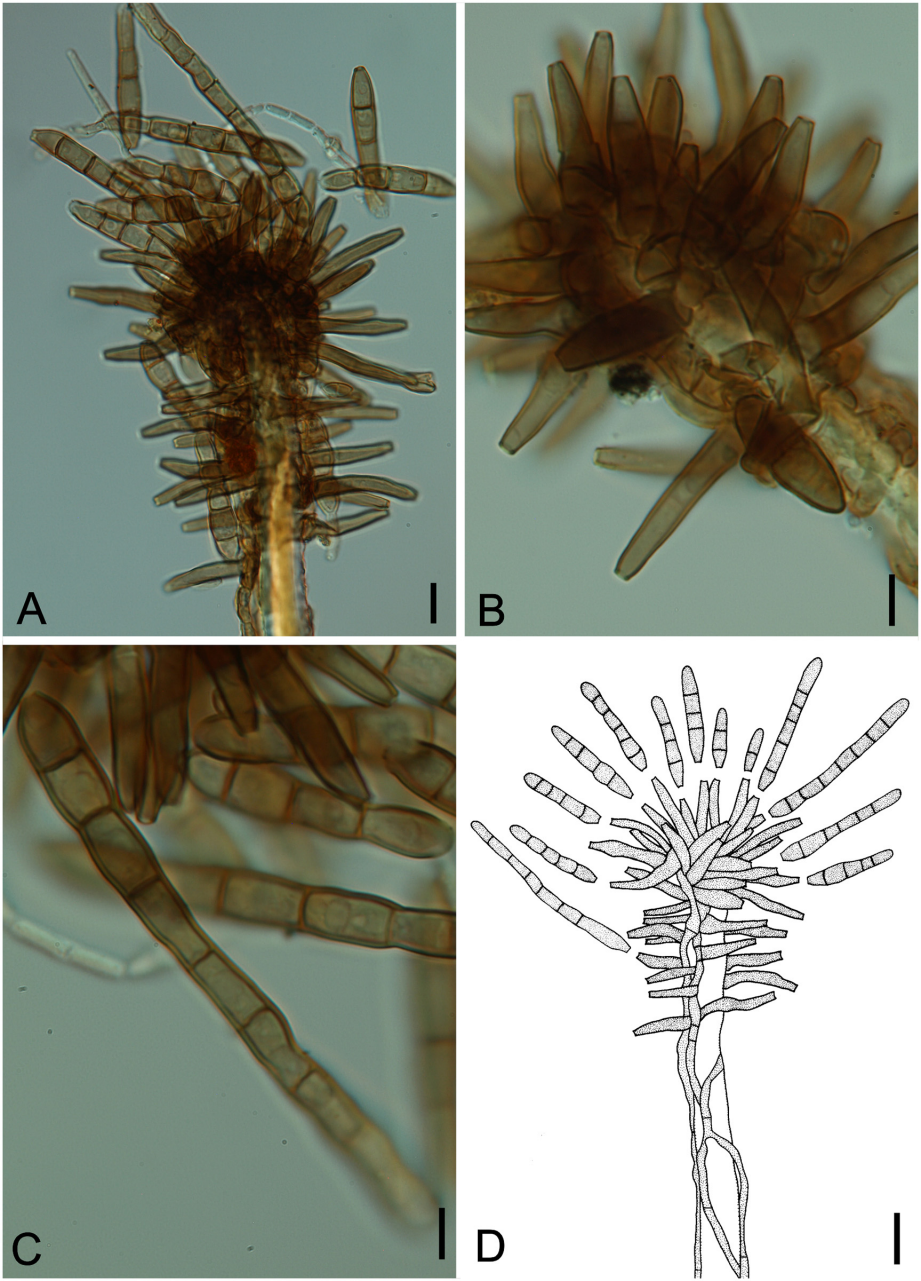
*Janetia wilsoniae* on *Dimorphandra wilsonii*. (A) Colonies on apex of trichome. (B) Monoblastic conidiogenous cells. (C) Euseptate, reddish brown conidia. (D) Conidiogenous cells and conidia. Bars: 10 μm (A); 5 μm (B,C).

*Janetia wilsoniae* M. Silva & R.W. Barreto, sp. nov. Fig 6

[urn:lsid:mycobank.org:names: 810522]

*Etymology*. In reference to the specific epithet of the host *Dimorphandra wilsonii*

Colonies hypophyllous, dense, brown, forming dark heads on trichomes. Internal mycelium indistinct. External mycelium superficial, up to 4 μm diam, branched, septate, brown, smooth. Conidiophores forming sporodochial clusters on the apex of trichomes, micronematous, mononematous. Conidiogenous cells holoblastic, monoblastic, cylindrical to narrowly ampulliform, straight to slightly curved, 7.5–37.5 × 2.5–5 μm, reddish brown to pale brown. Conidiogenous loci apical on conidiogenous cells, flat, truncate, not darkened, unthickened, conidial secession schizolytic. Conidia dry, solitary, obclavate to cylindrical, straight to slightly curved, occasionally slightly constricted at some of the septae, 12.5–75 × 5–7.5 μm, rounded at the apex up to 2.5 μm wide, rounded at the base 5 μm, 1–9 euseptate, hilum inconspicuous, guttulate, reddish brown to pale brown, smooth.

*In culture*: slow-growing (3.7–4.5 cm diam, after 27 days), chrysanthemoid to subcircular, flat, to convex, cottonose aerial mycelium accompanied by immersed growth within medium, dense, or without aerial mycelium and internal mycelium irradiating and sinuose and raising from the medium on radial strands, greenish centrally glaucous, followed by a periphery of pale olivaceous buff thick ring of mycelium, or olivaceous grey with pronounced diurnal zonation (under alternating light but less pronounced in the dark), bluish reverse, no sporulation.

*Specimens examined*: On living leaves of *Dimorphandra wilsonii*. BRAZIL: Minas Gerais: Paraopeba, Fazenda Tabuleiro Grande, 13 Jul 2009, M. Silva (VIC 31772 − HOLOTYPE); 19 Jul 2010, M. Silva (VIC 31780); 19 Jul 2010, M. Silva (VIC 31781).

*Notes*: The genus *Janetia* M. B. Ellis has 20 species that are characterized by the production of euseptate or distoseptate, phragmosporous conidia with schizolytic secession formed on pigmented, denticulate, monoblastic or polyblastic, integrated conidiogenous cells [30, 31, 32, 33, 34, 35] (Table 3). The two new species *Janetia wilsoniae* and *Janetia dimorphandra-mollis* have significantly different morphology. *Janetia wilsoniae* has longer conidiogenous cells than the latter and also has euseptate, smooth, longer and narrower conidia. *Janetia dimorphanda-mollis* is somewhat similar to *J*. *canensis* B. Sutton & Pascoe, described from the stems of *Acacia fimbriata* A. Cunn. ex Don and *A*. *linifolia* (Vent.) Willd (Fabaceae), *Janetia bacilliformis* Gamundí, Arambi & Giaiotti, found on leaves of *Nothofagus dombeyi* (Mirb.) Oerst. (Nothofagaceae), and *Janetia garryae* (Bonar) S. Hughes, which was described from the leaves of *Garrya fremontii* Torr. (Garryaceae). Nevertheless, *Janetia canensis* can be distinguished from *Janetia dimorphanda-mollis* by the presence of polyblastic conidiogenous cells, conidial walls that are deeply invaginated at the distosepta, smooth conidial walls and longer conidia (16–57 μm) [36]. *Janetia bacilliformis* differs from *Janetia dimorphanda-mollis* by having longer bacilliform conidia (60–156 μm long), monoblastic or polyblastic conidiogenous cells that are longer and wider (10–22 × 3–5 μm) and smooth conidial walls. *Janetia garryae* differs from *J*. *dimorphanda-mollis* by having euseptate and longer conidia (25–70 μm long) [31]. Hence, the introduction of a new species is justified for *J*. *dimorphanda-mollis*.

**Table 3:** Conidial size and septation of *Janetia* species recorded on members of the *Fabaceae*.

Species	Conidial size μm	Septation
*J*. *bacilliformis* Gamundí, Arambi & Giaiotti	60–156 × 5–9	5–9 distoseptate
*J*. *canescens* B. Sutton & Pascoe	16–57 × 5.5–9	1–7 distoseptate
*J*. *refugia* B. Sutton & Pascoe	31–37 × 7–8	4–6 distoseptate
*J*. *synnematosa* Sivan. W.H. Hsieh	80–115 × 10–12.5	9–22 distoseptate
***Janetia dimorphandra-mollis***	**16–35 × 6.5–9.5**	**2–7 distoseptate**
*J*. *bonarii* (M.B. Ellis) S. Hughes	55–95 × 10–12	5–12 euseptate
*J*. *capnophila* S. Hughes	58–145 × 10.8–6.2	7–16 euseptate
*J*. *cubensis* Matsush.	16–77 × 5–8	2–8 euseptate
*J*. *curviapisis* Goh & K.D. Hyde	65–100 × 5.5–7.5	6–12 euseptate
*J*. *euphorbiae* M.B. Ellis	18–36 × 6–8	3–6 euseptate
*J*. *faureae* (Piroz.) M.B. Ellis	50–120 × 4–5	3–9 euseptate
*J*. *garryae* (Bonar) S. Hughes	25–70 × 6–8.5	2–6 euseptate
*J*. *indica* S.R.Bussa	65–105 × 9–15	5–9 euseptate
*J*. *interna* H.J. Swart	57–128 × 10–11	5–8 euseptate
*J*. *leprosa* (Piroz.) S. Hughes	10–17 × 3.5–4	2–3 euseptate
*J*. *longispora* P.M. Kirk	90–285 × 10–15	6–12 euseptate
*J*. *mangiferae* S. Hughes & Cavalc.	8.5–23 × 4.3–6	1–5 euseptate
*J*. *matsushimae* Subram.	20–31.5 × 5–6	4–7 euseptate
*J*. *obovata* M. Calduch, Gené, Abdullah & Guarro	22.5–33.5 × 12–15	3–5 euseptate
*J*. *salvertiae* Dorn.-Silva & Dianese	15–30 × 3–5	1–6 euseptate
*J*. *salicis* Li Xu & Y.L. Guo	34–91 × 2.5–4	multiseptate
***Janetia wilsoniae***	**12.5–75 × 5–7.5**	**1–9 euseptate**

*Janetia wilsoniae* is similar to *Janetia salvertiae* Dornelo-Silva and Dianese, which occurs on *Salvertia convallariodora* St. Hil. (Vochysiaceae) and *Vochysia* sp. (Vochysiaceae), *Janetia euphorbiae* M. B. Ellis, which colonizes the stems of *Euphorbia tirulicallis* L. (Euphorbiaceae), and *J*. *cubensis* Matsush., which was described from the leaves of *Roystoneae regiae* (Kunth) O.F. Cook (Arecaceae). *Janetia salvertiae* differs from *J*. *wilsoniae* by having clavate, shorter and narrower conidia (15–30 × 3–5 μm) [34]. *Janetia euphorbiae* is distinguished from *Janetia wilsoniae* by having shorter and wider conidia (18–36 x 6–8 μm) and not forming sporodochial groups on trichomes in contrast to the new species [30]. *Janetia cubensis* has conidia with a morphology which is similar to that of *Janetia wilsoniae*. Nevertheless, Goh & Hyde [32] suggested that it is unlikely that *J*. *cubensis* belong to *Janetia* because it has rhexolytic conidial secession and its conidiogenous “denticles” do not appear to be bulbous [32, 35]. Therefore, *Janetia wilsoniae* cannot be adequately placed in any known species of *Janetia* and is described here as a new species.

Only *Janetia wilsoniae* was successfully isolated in a pure culture. The investigation of sequences obtained from that taxon indicated that it grouped closely with *Zasmidium cellare* (Pers.) Fr., *Z*. *nocoxi* Crous, *Z*. *xenoparkii* (Crous & M.J. Wingf.) Crous & U. Braun, *Mycospaherella aleuritidis* (I. Myiake) S.H. Ou and *Ramichloridium cerophilum* (Tubaki) de Hoog in a clade that was highly supported in the family Mycosphaerellaceae. The lack of any other sequences of a member of *Janetia* in sequence databases and the lack of knowledge of its sexual morph connection limits the understanding of the phylogenetic relationships within this taxon (Fig 2).

*Johansonia chapadiensis* Crous, R.W. Barreto, Alfenas & R.F. Alfenas, *IMA Fungus* 1: 117–122 (2010).

Descriptions and illustrations––Crous et al. (2010: 117–122, Figure 120).

*Specimens examined*: On living leaves of *Dimorphandra mollis*. BRAZIL: Minas Gerais: Paraopeba, Flona, 19 Jul 2010, *M*. *Silva* & *O*.*L*. *Pereira* (VIC 31779); on living leaves of *Dimorphandra wilsonii*. BRAZIL: Minas Gerais: Paraopeba, Fazenda Tabuleiro Grande, 25 Jul 2011, *M*. *Silva* & *O*.*L*. *Pereira* (VIC 31795).

*Microcalliopsis dipterygis* Batista, Peres & Bezerra, Brotéria Série de Ciências Naturais, 31: 1–26 (1962) Figs 7 and 8

**Fig 7:**
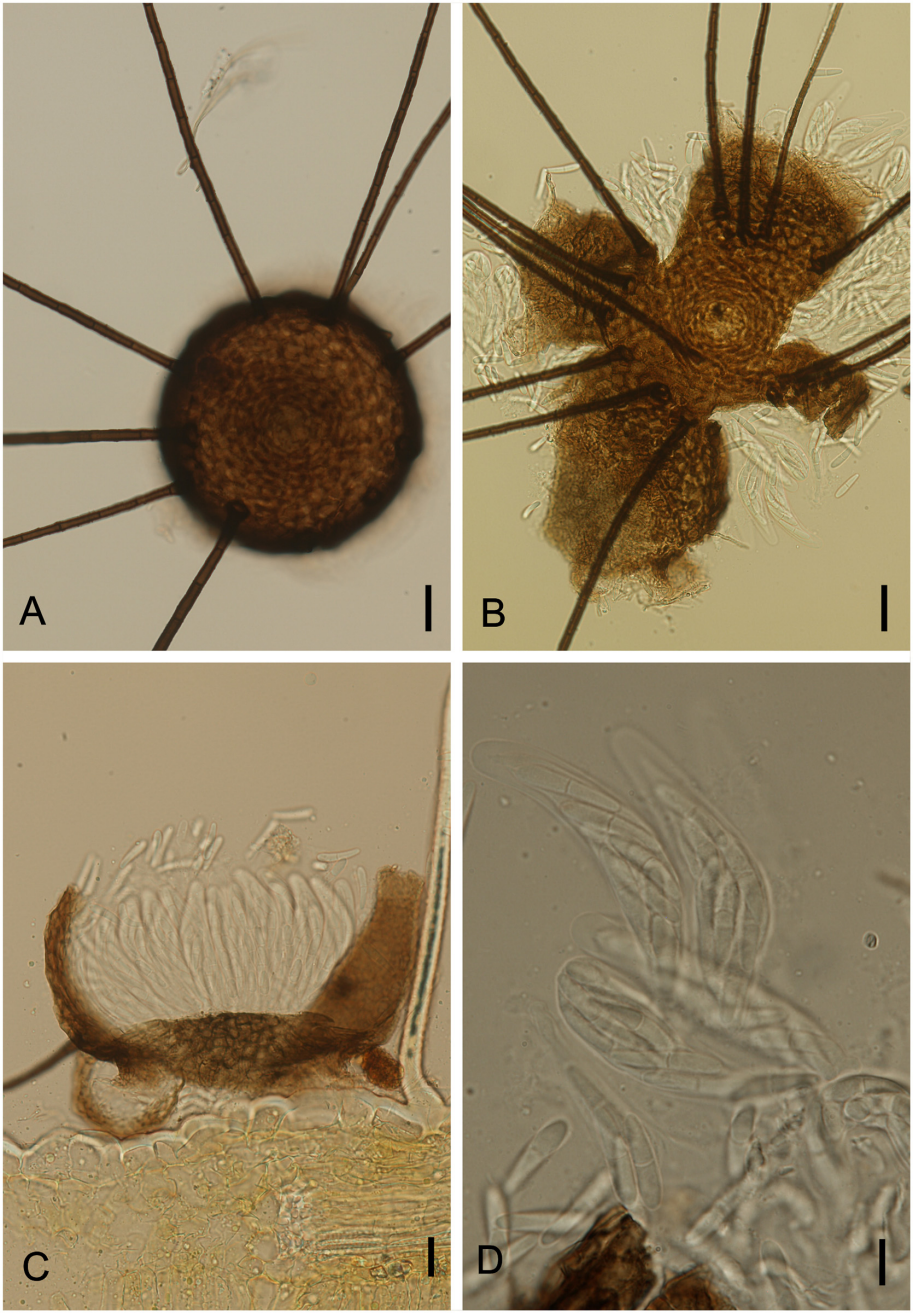
*Microcalliopsis dipterygis* on *Dimorphandra wilsonii*. (A) Upper view of pseudothecial ascoma bearing needle-shaped setae c. (B) Squashed ascoma releasing asci and ascospores. (C) Cross section of pseudothecium showing parallel asci. (D) Bitunicate asci with 1-septate hyaline ascospores. Bars: 20 μm (A, B, C); 10 μm (D).

**Fig 8:**
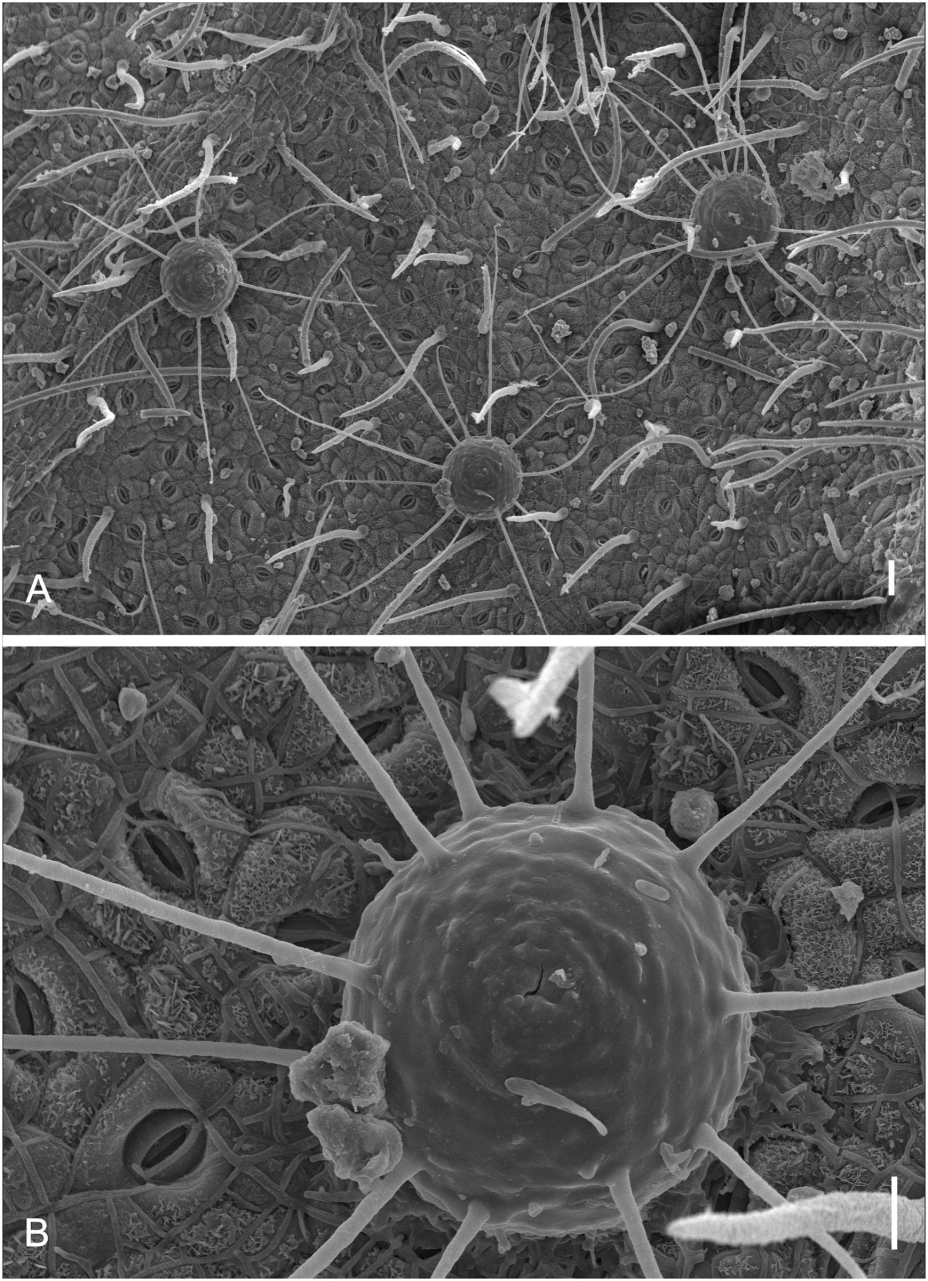
SEM of *Microcalliopsis dipterygis* on *Dimorphandra wilsonii*. (A) Ascomata attached to leaf surface. Note sputnik-like shape of pseudothecia (B) Detail of inconspicuous ostiole, protuberances on external wall and setae on individual ascoma. Bars: 50 μm (A); 20 μm (B).

Colonies on living leaves, adaxial, sooty, irregular, sparse. Internal mycelium indistinct. External mycelium hypogenous, 2.5–3 μm diam, branched, net-forming, slightly undulate, composed of septate, brown, smooth hyphae. Ascomata pseudothecial, hypophyllous, superficial, solitary, ostiolate, irregularly scattered over the surface of the colonies, spherical to somewhat flattened, oblate spheroidal, 120–170 × 58–90 μm, walls of brown textura angularis, 10–17.5 μm thick, smooth. Setae 6–9 per ascoma, evenly arising mostly from the lower half of the pseudothecia, needle-shaped mostly straight to slightly curved, 140–600 × 3–5 μm, multiseptate (up to 22), smooth, dark brown, unbranched, tips rounded. Asci bitunicate, parallel, obclavate, 57.5–62.5 × 12.5–15 μm, 8–spored, endotunica extending as a narrow column into the conspicuously domed ascal apex. Ascospores inordinate, fusiform to ellipsoid, with rounded ends, 17.5–22.5 × 4–5 μm, 1–septate, eguttulate, hyaline, smooth.

*Specimens examined*: On living leaves of *Dimorphandra wilsonii*. BRAZIL, Minas Gerais: Paraopeba, Fazenda Tabuleiro Grande, 27 Jul 2011, M. Silva (VIC 31806).

*Notes*: J. L. Bezerra examined our material and recognized it as a taxon described by him more than fifty years ago. The fungus on *D*. *wilsonii* belongs to the obscure genus *Microcalliopsis* Bat. & Cif. (Chaetothyriaceae) [37]. There are only four known species of *Microcalliopsis* and our specimens fitted well within the description of *M*. *dipterygis*, which was previously reported on the leaves of *Dipterys alata* Vog. in Brazil [37]. The occurrence of this fungus on *D*. *wilsonii* suggests that it can be a polyphagous fungus on the leaves of leguminous hosts. This is the first record of *Microcalliopsis dipterygis* colonizing *D*. *wilsonii*.

*Phillipsiella atra* Cooke, *Grevillea* (1878) Fig 9

**Fig 9:**
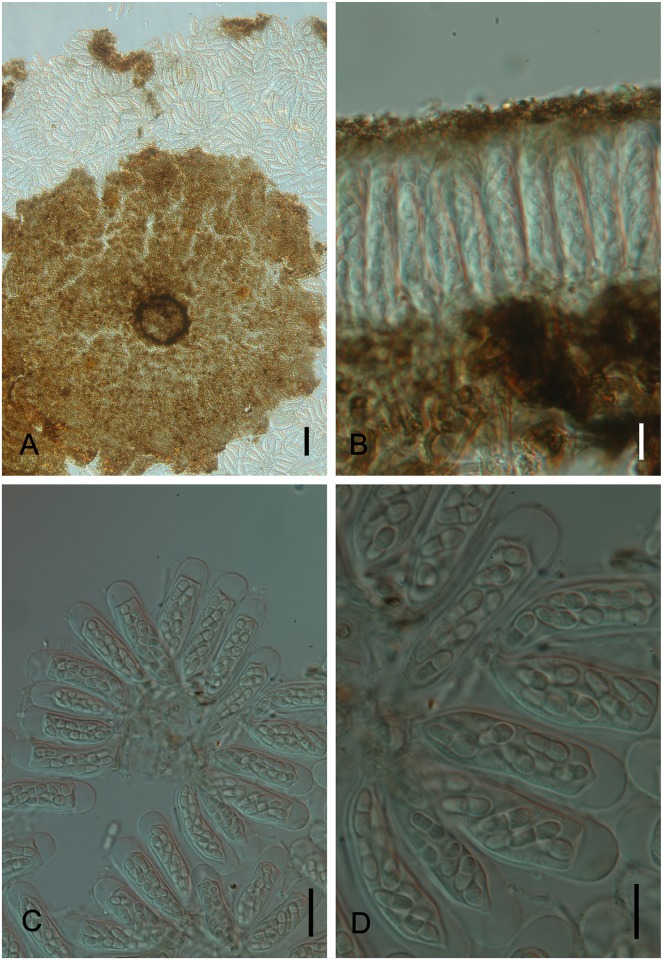
*Phillipsiella atra* on *Dimorphandra wilsonii*. (A) Apothecioid ascomata attached to the abaxial side of a leaf. (B) Squash mount of discoid ascoma. (C) Cross section of fertile ascoma showing parallel asci. (D) Bitunicate asci containing eight, 1-septate, hyaline ascospores. Bars: 50 μm (B); 10 μm (C, D).

Colonies on living leaves, hypophyllous, sparsely spread, not associated with disease symptoms on host. Internal mycelium not observed. External mycelium hypogenous, 2.5–4 μm diam, branched, net-forming, slightly undulate, composed of septate, pale brown, smooth hyphae. Ascomata apothecioid, hypophyllous, superficial, solitary, discoid, non-ostiolate, 445–550×75–105 μm high, pseudoparenchymatous basal stroma 12.5–20 thick, walls of brown textura angularis, powdery, ill-differentiated, dark grey centrally with raised pale gray margins, pseudoparaphyses filiform, up to 2 μm wide, septate, unbranched, hyaline, but branching above the asci, becoming inflated and pigmented at apices to form an epithecium. Asci bitunicate, parallel, cylindrical, 30–40 × 7.5–11μm, 8–spored, endotunica flattened apically at a distance from apical exotunica but extending as a narrow column towards the conspicuously domed ascal apex. Ascospores biseriate to inordinate, fusiform to ellipsoid, 7.5–10 × 2.5–4 μm, 1–septate, upper cell slightly broader than lower cell, eguttulate, hyaline, smooth.

*Specimens examined*: On living leaves of *Dimorphandra wilsonii*. BRAZIL: Minas Gerais: Paraopeba, Fazenda Tabuleiro Grande, 14 Jul 2011, M. Silva (VIC 31773); 27 Jul 2011, M. Silva (VIC 31805); on living leaves of *Dimorphandra wilsonii*. BRAZIL: Minas Gerais: Caetanópolis, Fazenda São Bento, 27 Jul 2011, M. Silva (VIC 31807).

*Notes*: The genus *Phillipsiella* was proposed by Cooke [38] with *Phillipsiella atra* as the type species was collected on *Quercus virginiana* Mill (Fagaceae) in Georgia [39]. Key features for the genus were: discoid ascomata containing numerous asci, asci bitunicate and hyaline, bicellularascospores. There are twelve species accepted within this genus, but none of these species has been reported in association with members of the Fabaceae [40]. Our specimens collected on *D*. *wilsonii* are the first to be reported growing on a member of the Fabaceae, and they fitted well within the description of *P*. *atra*. Müller and von Arx [41] placed the genus *Phillipsiella* within Schizothyriaceae. Later, von Arx & Müller [23] transferred *Phillipsiella* to the Saccardiaceae. Posteriorly, Barr [42] revived the family Phillipsiellaceae of von Höhnel [43], and Eriksson [44] also discussed and accepted the Phillipsiellaceae [45]. However, the recent publication on the classification of families of Dothideomycetes does not discuss the relationship of Saccardiaceae and Phillipsiellaceae [46]. Based on the analysis of large ribosomal subunit DNA gene sequences and the resulting phylogeny generated in the present study (Fig 2), it is finally clarified that *Phillipsiella* belongs to the Dothideomycetes (Capnodiales). Another fungus (*Johansonia chapadiensis* Crous, R.W. Barreto, Alfenas & R.F. Alfenas) was recently described from leaves of *Dimorphandra mollis* collected in Chapada dos Guimarães, Mato Grosso, Brazil. The phylogenetic study based on DNA sequence data of the nuclear ribosomal DNA (LSU) showed that *Johansonia* is also a member of *Dothideomycetes*, *Capnodiales* [47]. The fact that *Phillipsiella* and *J*. *chapadiensis* are grouped in the same highly supported clade (Fig 2) indicates that these two genera belong to the same family (possibly the Saccardiaceae). Unfortunately, there is no molecular information for the type species of the family Saccardiaceae which may be used for a clarification of the affinities of these taxa. Therefore, it is not possible to circumscribe the Saccardiaceae adequately, and at present it is also impossible to confirm that these genera belong to this family as proposed by von Arx & Müller [27].

*Piricauda paraguayensis* (Speg.) R. T. Moore, *Mycologia* 50: 691 (1959) Figs 10 and 11

**Fig 10:**
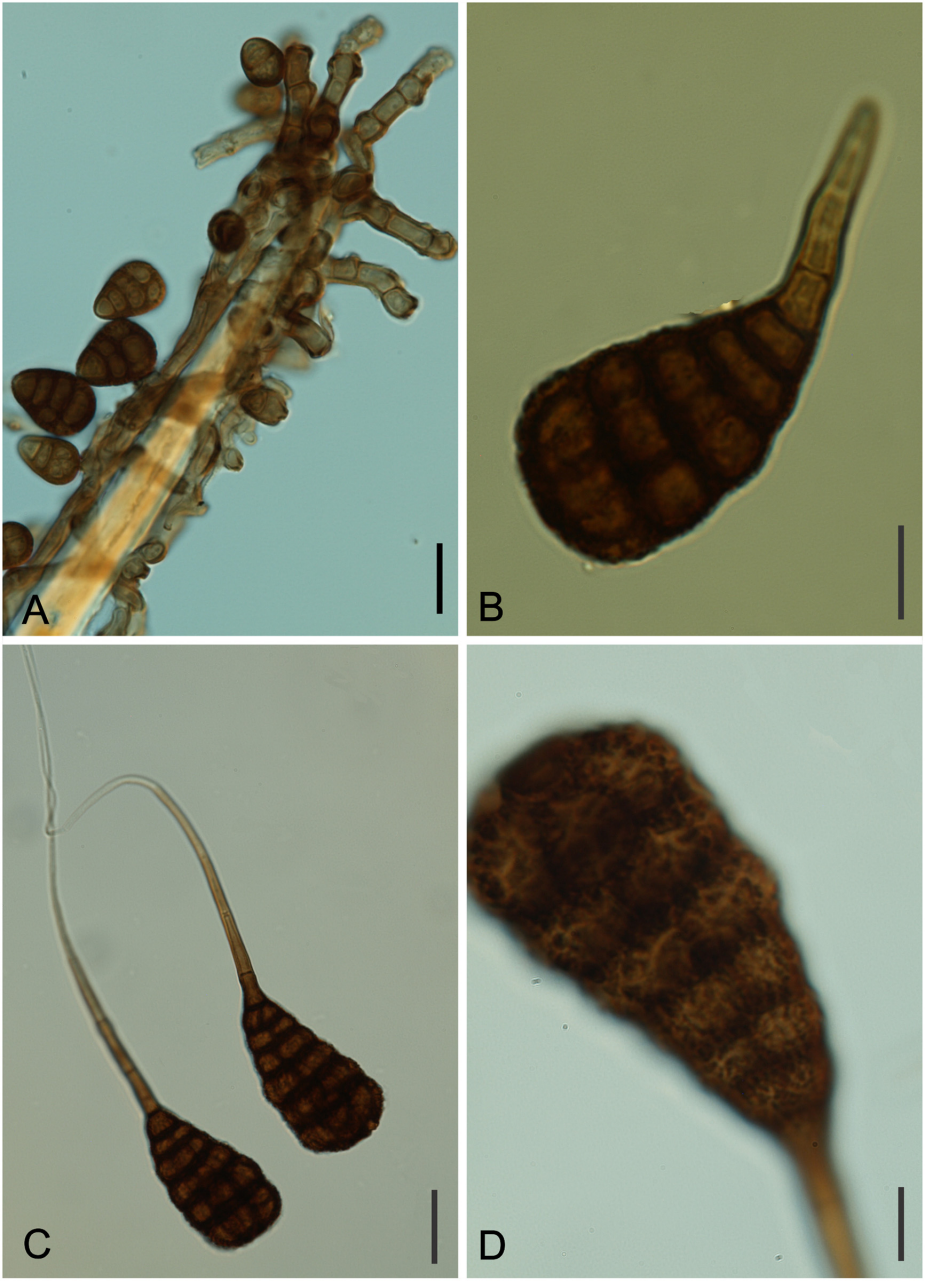
*Piricauda paraguayensis* on *Dimorphandra wilsonii*. (A) Colony with immature conidia (notice the absence of beaks) on trichomes. (B) Dictyosepatate conidium developing beak. (C) Mature conidia with fully developed beak. (D) Close-up of mature conidium showing irregular rugose (with seemingly reticulate patter) surface. Bars: 20 μm (A, C); 10 μm (B, D).

**Fig 11:**
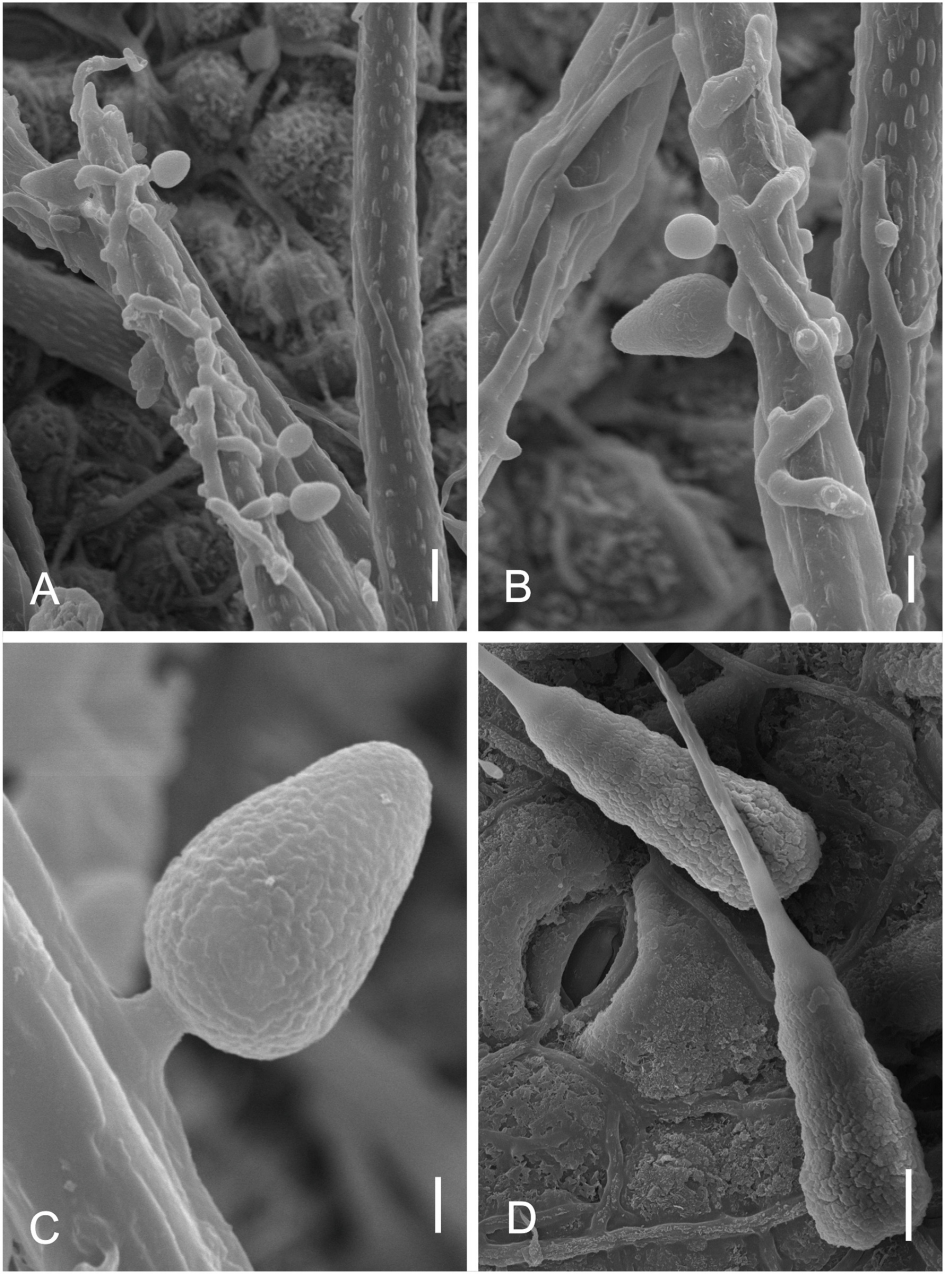
SEM of *Piricauda paraguayensis* on *Dimorphandra* spp. (A) Colonies on trichome. (B) *Ibid* closer view with conidiogenous cell and conidia at early stages of development. (C) Close-up of immature conidium on conidiogenous cell. (D) Mature conidia on leaf surface (note smooth beaks and irregularly rugose bodies). Bars: 10 μm (A, D); 5 μm (B); 2 μm (C).

Colonies hypophyllous, effuse, black, on trichomes. Internal mycelium indistinct. External mycelium superficial, 2.5–4 μm, branched, septate, grayish brown, smooth. Conidiophores on trichomes, micronematous, formed along the hyphae, sometimes macronematous terminally at the apex of trichomes, mononematous, cylindrical, 5–62.5 × 5.0–7.5 μm, 0–5 septate, occasionally branched, brown, smooth. Conidiogenous cells terminal or intercalary, integrated, monotretic, cylindrical, 10–25× 5–7.5 μm, light brown. Conidiogenous loci often with conspicuous dark scars with a well-defined pore in the middle, 1–6 per cell, up to 2.5 μm diam. Conidia dry, solitary, ovoid becoming pyriform, beaked at maturity, 15–20 × 10–12.5 when immature becoming 32.5–60 × 17.5–30 μm (body) when mature, dictyoseptate, 5–6 transversally and 4–7 longitudinal septa, pale brown to dark brown, rugose, beak, 40–125 × 2.5–4.5 μm, brown to pale brown to subhyaline terminally, tapering to 1.5–2.5 diam at apex, 3–5 septate.

*In culture*: slow-growing (0.6–1.6 cm diam, after 27 days), either of scanty floccose aerial mycelium and growing very poorly or of mostly immersed lobate, flat, dentritic mycelium, colony composed of monilioid and filamentous hyphae, striate and granulose, dark mouse gray, to iron gray, reverse dark olivaceous with slight yellow pigmentation of medium, no sporulation.

*Specimens examined*: On living leaves of *Dimorphandra mollis*. BRAZIL: Minas Gerais: Paraopeba, Floresta Nacional de Paraopeba, 20 Jul 2010, M. Silva (VIC 31782); on living leaves of *Dimorphandra wilsonii*. Brazil, Minas Gerais: Paraopeba, Fazenda Tabuleiro Grande, 21 Jul 2010, M. Silva (VIC 31785).

*Notes*: The morphology of fungi in the genus *Piricauda* Bubák according to the generic concept of Hughes [48] and Ellis [49] is micronematous conidiophores developing on superficial hyphae, conidiogenous cells monotretic and conidia tretic arising singly from a pore on the conidiogenous cell. There are eight species in the genus *Piricauda*: *P*. *cochinensis* (Subram.) M. B. Ellis, *P*. *cubensis* Hol.Jech. & Mercado, *P*. *longispora* Mercado, Guiné & Guarro, *P*. *mexicana* Mercado, Heredia & Mena, *P*. *paraguayensis* (Speg) R.T. Moore, *P*. *pseudarthriae* (Hansf.) M.B. Ellis, *P*. *taiwanensis* Matsush. and *P*. *vulcanensis* on several hosts [30, 49, 50]. Our species fits well within the boundaries of *P*. *paraguayensis* as described by Ellis [48]. This species was previously reported on *Bignonia* sp., *Citharexylum* sp. and *Duranta* sp. in Brazil, Cuba and Paraguay [49, 50]. This is the first report of this fungus on *D*. *wilsonii* and *D*. *mollis*. Phylogenetically, *P*. *paraguayensis* grouped in a clade in the Capnodiales, Mycosphaerellaceae (Fig 2). This is the first report of this fungus in culture. More sequences of species of *Piricauda* are necessary to better elucidate the phylogenetic position of this obscure genus.

**Fig 12:**
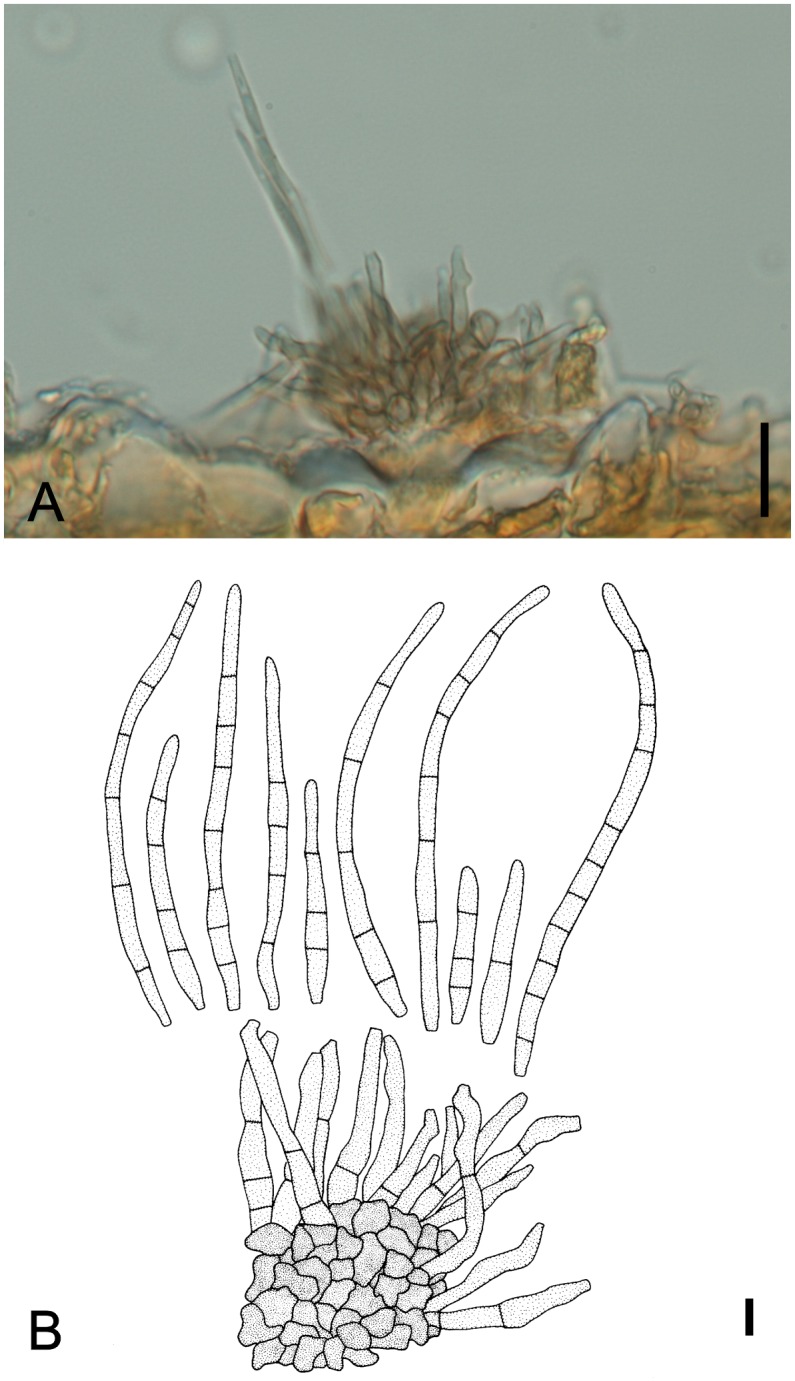
*Pseudocercospora dimorphandae* on *Dimorphandra* spp. (A) Cross section of substomatal cavity from which conidiophores emerge. (B) Conidiophores both organized in a sporodochium and multiseptate conidia. Bars: 20 μm (A); 10 μm (B).

*Pseudocercospora dimorphandrae* M. Silva & R.W. Barreto, sp. nov. Fig 12

[urn:lsid:mycobank.org:names: 810524]

*Etymology*. In reference to host genus *Dimorphandra*

Lesions on living leaves amphigenous, starting as chlorosis that later develop into necrosis of oldest parts of leaves, irregular, brown, 1.5–8 mm diam, coalescing to encompass entire leaflets and leading to leaf blight. Internal mycelium indistinct. External mycelium absent. Stromata well developed, substomatal, irregular to convex, 25–40 × 30–62.5 μm. Conidiophores hypophyllous arising from stromata, in sporodochia, cylindrical—obclavate, straight to curved or sinuous, 10–27.5 × 2.5–5 μm, 0–3 septate, unbranched, light brown, smooth, mostly restricted to the conidiogenous cells. Conidiogenous cells terminal, holoblastic, integrated, mostly cylindrical, light brown. Conidiogenous loci terminal, inconspicuous, truncate, 1–2.5 μm diam, neither thickened nor darkened. Conidia dry, solitary, cylindrical, mostly curved, 17.5–87.5 × 3–4 μm, truncate at the base, tapering towards a subacute apex, 1–12 septate, hilum unthickened and not darkened, subhyaline to olivaceous, guttulate, smooth.

*In culture*: On PCA slow-growing (2.1–2.8 cm diam, after 27 days), slightly to pronouncedly lobate at edges, flat to low convex and pale olivaceous grey cerebriform centrally, surrounded with a ring of honey colored immersed mycelium followed by a narrow ring of olivaceous mycelium, followed by a periphery of pale olivaceous grey mycelium, diurnal zonation either pronounced or subtle, reverse leaden black followed by a periphery of leaden gray mycelium; not sporulating.

*Specimens examined*: On living leaves of *Dimorphandra wilsonii*. BRAZIL: Minas Gerais: Paraopeba, Fazenda Tabuleiro Grande, 14 Jul 2009, M. Silva (VIC 31774− HOLOTYPE); 25 Jul 2011, M. Silva (VIC 31797); on living leaves of *Dimorphandra mollis*. BRAZIL: Minas Gerais: Paraopeba, Floresta Nacional de Paraopeba, 15 Jul 2009, M. Silva (VIC 31775).

*Notes*: *Pseudocercospora* Speg. is one of the largest genera of fungi and includes more than 1200 species [51]. Approximately 200 are parasitic on members of the Fabaceae. Cercosporoid fungi bearing unthickened and not darkened conidiogenous loci and hila and having pigmented conidiophores and conidia are often placed in *Pseudocercospora* [52, 53, 54]. The fungus on *Dimorphandra* has the typical morphological features of members of *Pseudocercospora* and was compared with species of *Pseudocercospora* reported on hosts phylogenetically close to *Dimorphandra*, such as the *Dimorphandra*-group (Banks and Lewis 2009) including *Burkea* Benth., *Erythrophleum* Afzel. Ex R. Br., *Mora* Schomb. ex Benth., *Pachyelasma* Harms, *Stachyothyrsus* Harms and *Sympetalandra* Stapf. Only one species of *Pseudocercospora* is known to occur on a member of this group (*Pseudocercospora erythrophlei* Z.Q. Yuan reported on the leaves of *Erythrophleum chlorostachys* Baill) [55]. This is the first report of *Pseudocercospora* on a member of *Dimorphandra*, and the fungus found in this study clearly differs from *P*. *erythrophlei* by having shorter conidiophores and conidia (10–27 μm and 17.5–87.5 μm, respectively) and not having geniculate conidiogenous cells with short “denticles” as found in *P*. *erythrophlei*, thereby justifying the proposition of the new species. The morphology of specimens of *Pseudocercopora* on *D*. *wilsonni* and on *D*. *mollis* is identical. A comparison of the DNA sequences obtained from both species confirmed that the isolates obtained from the two hosts belonged to the same species (Fig 2). The *Pseudocercospora* on *Dimorphandra* spp. grouped in the same clade of the type species of the genus *Pseudocercospora* (*P*. *vitis*) and it is phylogenetically close to *Mycosphaerella bixae* Crous & Bench. *Mycosphaerella bixae* is a parasite of a distantly related host (*Bixa orellana* L.) belonging to the family Bixaceae and has a *Passalora* asexual morph Crous & Bench [52, 56]. Additionally, sequences of the ITS region of *Pseudocercospora dimorphandrae* have only 97% of similarity with the ITS sequence of *P*. *bixae* deposited in GenBank (Accession No. AF362056).

**Fig 13:**
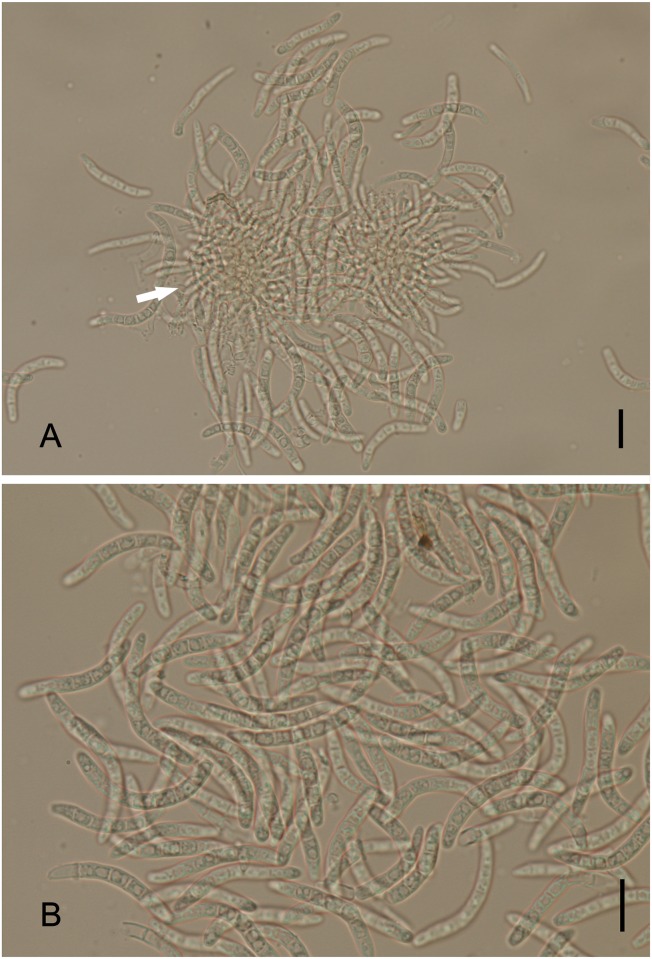
*Pseudocercosporella dimorphandae* on *Dimorphandra* spp. (A) Squashed mount showing group of unbranched conidiophores (arrowed) and conidia. (B) Close-up of curved conidia. Bars: 20 μm.

*Pseudocercosporella dimorphandrae* M. Silva & R.W. Barreto, sp. nov. Fig 13

[urn:lsid:mycobank.org:names: 810525]

*Etymology*: In reference to the genus of the host *Dimorphandra*

Lesions on living leaves amphigenous, starting as minute dark dots becoming circular to irregular, necrotic, coalescing and leading to leaf blight, 1.5–5 mm diam, brown, covered with white foamy-like fungal colonies, coalescing to cover the whole surface of the leaflets and leading to leaflet blight. Internal mycelium inter and intracellular, 1.5–2.5 μm, branched, septate, pale brown. External mycelium absent. Stromata superficial, 17.5–30 × 37.5–55 μm, composed of subhyaline textura angularis. Conidiophores mostly restricted to conidiogenous cells, hypophyllous arising from stromata, in dense fascicles, cylindrical, straight to slightly sinuous, 10–20 × 2.5–5 μm, 0–1 septate, unbranched, hyaline, smooth,. Conidiogenuous cells integrated, terminal, holoblastic, cylindrical, hyaline. Conidiogenous loci, truncate, up to 2.5 μm diam, neither thickened nor darkened. Conidia dry, solitary, cylindrical, slightly curved to curved, 22.5–57.5 × 4–6 μm, truncate at the base, apex rounded, 2–7 septate, hilum unthickened and not darkened, hyaline to olivaceous, guttulate, smooth.

*In culture*: On PCA slow-growing (2–3 cm diam, after 27 days), slightly lobate edges, flat, immersed on media at periphery, aerial mycelium buff cottonous either followed with an outer ring with irregular portions of buff or honey cottonose mycelium or followed by a narrow ring of white cottonous mycelium over a layer of olivaceous gray colony, followed by a periphery of pale olivaceous gray mycelium, reverse either rosy buff or primrose alternating with leaden black; not sporulating.

*Specimens examined*: On living leaves of *Dimorphandra wilsonii*. BRAZIL: Minas Gerais: Paraopeba, Fazenda Tabuleiro Grande, 26 Jul 2011, M. Silva (VIC 31788− HOLOTYPE); 26 Jul 2011, M. Silva (VIC 31798, on living leaves of *Dimorphandra mollis*. BRAZIL: Minas Gerais: Paraopeba, Floresta Nacional de Paraopeba, 15 Jul 2009, M. Silva (VIC 31776); 20 Jul 2010, M. Silva (VIC 31783); 08 Feb 2011, M. Silva (VIC 31789); on living leaves of *Dimorphandra wilsonii*. BRAZIL: Minas Gerais: Juatuba, 07 Feb 2011, M. Silva (VIC 31786); on living leaves of *Dimorphandra wilsonii*. BRAZIL: Minas Gerais: Fortuna de Minas, 07 Feb 2011, M. Silva (VIC 31787); 08 Feb 2011, M. Silva (VIC 31793); on living leaves of *Dimorphandra wilsonii*. BRAZIL: Minas Gerais: Sete Lagoas, 09 Feb 2011, M. Silva (VIC 31790); 09 Feb 2011, M. Silva (VIC 31791); 09 Feb 2011, M. Silva (VIC 31792).

*Notes*: The cercosporoid fungus found on *D*. *wilsonii* and *D*. *mollis* clearly belongs to the genus *Pseudocercosporella* Deighton. It bears the key morphological characteristics of the genus: hyaline conidiophores, bearing unthickened and not darkened scars, hyaline conidia, bearing unthickened and not darkened hila and released by schizolytic secession [57]. Four *Pseudocercosporella* species are known to occur on members of the Fabaceae [40]: *P*. *astragali* (Rostr.) U. Braun, *P*. *cystisi* (Jaap) U. Braun, *P*. *ougeiniae* M.D. Mehrotra & R.K. Verma and *P*. *tephrosiae* (Hansf.) U. Braun. *Pseudocercosporella astragali* and *P*. *cystisi* are easily distinguished from *Pseudocercosporella* on *Dimorphandra* by having longer and narrower conidia (15–70 × 2.5–4 μm and 50–125 × 1–4 μm, respectively). *Pseudocercosporella ougeiniae* has longer conidia [(35)60–90(115) μm] and narrower conidiophores (3–4 μm). *Pseudocercosporella tephrosiae* has longer conidiophores [50–100 (125) μm] and smaller conidia (15–45 μm) than the fungus on *Dimorphandra* [58, 59] (Table 4). The *Pseudocercosporella* on *Dimorphandra* spp. is phylogenetically close to *Mycosphaerella ellipsoidea* Crous & M.J. Wingf., *M*. *endophytica* Crous & H. Smith, *M*. *gregaria* Carnegie & Keane and *M*. *stromatosa* J. E. Taylor & Crous. *Mycosphaerella ellipsoidea* (≡*Amycosphaerella africana*) and *M*. *stromatosa* are known to have *Uwibraunia* Crous & M.J. Wingf. and *Pseudocercospora* as asexual morphs, respectively [60, 61, 62, 63], whereas no asexual morph is known for *M*. *gregaria* (≡ *Phaeophleospora gregaria*) [62, 63]. Only *M*. *endophytica* has a *Pseudocercosporella* asexual morph, but this species possesses a clearly distinct morphology. It has longer and narrower conidiophores (20–60 × 3–4 μm), smaller and narrower conidia (13–50 × 1.5–2.5 μm), and is a parasite of a member of a distantly related host family (*Eucalyptus*, Myrtaceae). Moreover, ITS region sequences from *Pseudocercosporella dimorphandrae* has 67, 41, 41 and 40 nucleotide differences as compared to *M*. *ellipsoidea*, *M*. *endophytica*, *M*. *gregaria* and *M*. *stromatosa*, respectively. Additionally, the significant level of host specificity known for this group of fungi provides further evidence that the fungus associated with *Dimorphandra* is a new species. The overlap in the DNA sequence analysis (Fig 2) and the equivalent morphology found for *Pseudocercosporella* specimens on *D*. *wilsonni* and *D*. *mollis* clearly shows that they belong to the same species.

**Table 4:** Conidia and conidiophore size of *Pseudocercercosporella* species recorded on members of the *Fabaceae*.

Species	Conidia size (μm)	Conidiophore size (μm)
*P*. *astragali* (Rostr.) U. Braun	15–70 x 2.5–4	4–10 x 3–4
*P*. *cystisi* (Jaap) U. Braun	50–125 x 1–4	10–40 2–5
*P*. *ougeniae* M.D. Mehrotra & R.K. Verma	(35)60-90(115) x 5–6.5	10–20 x 3–4.5(4)
*P*. *tephrosiae* (Hansf.) U. Braun	(15)15-45 x (3)4-5(6)	50-100(125) x 2.5–5.5
***Pseudocercosporella dimorphandrae***	**22.5–57.5 × 3.75–6.25**	**10–20 × 2.5–5.0**

**Fig 14:**
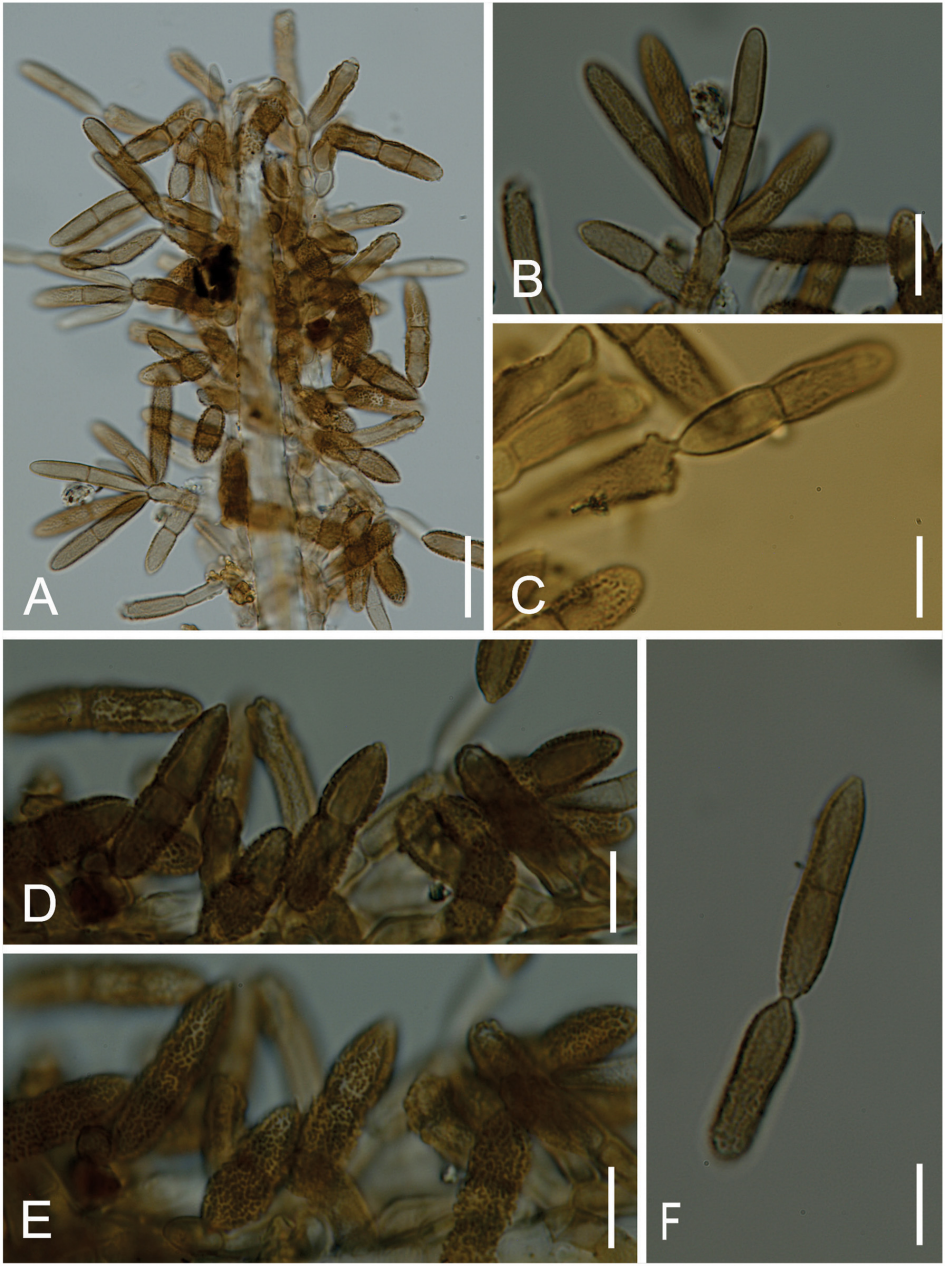
*Ramichloridiopsis wilsoniae* on *Dimorphandra wilsonii*. (A) Dense colony on trichome. (B) Conidiogenous cell bearing slightly darkened scars. (C) Close-up of semidenticulate conidiogenous cell. (D) Fusiform to cylindrical, septate mature conidia. (E) Different plane of focus showing verruculose longitudinal rows on conidiua. (E) Catenate conidia. Bars: 20 μm (A, B, C, D); 10 μm (E).

**Fig 15:**
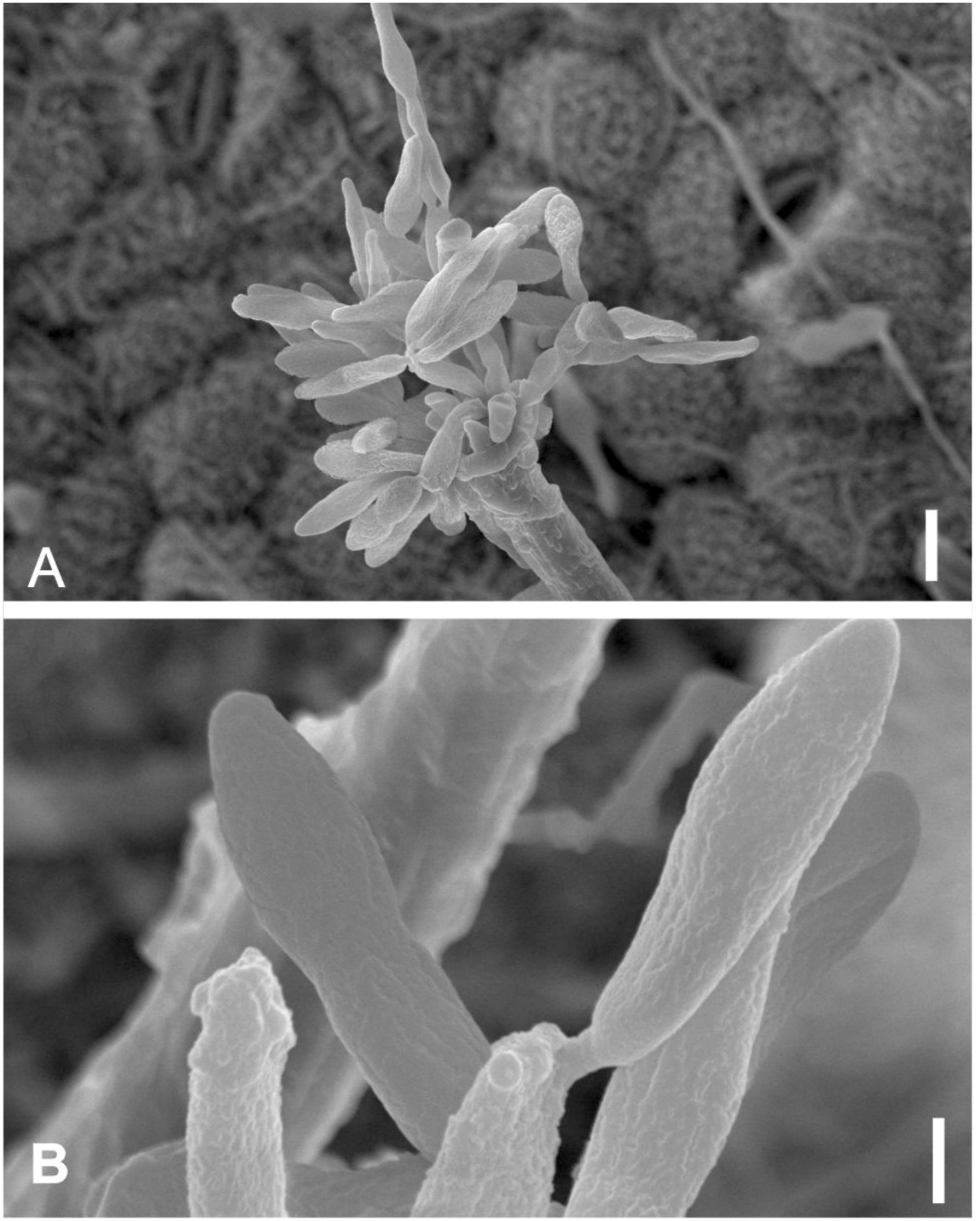
SEM of *Ramichloridiopsis wilsoniae* on *Dimorphandra wilsonii* (A) Colony on trichome (B) Terminal polyblastic, conidiogenous cells. Bars: 10 μm (A); 5 μm (B).

*Ramichloridiopsis* M. Silva & R.W. Barreto, gen. nov. Figs 14 and 15

[urn:lsid:mycobank.org:names: 810526]

*Type species*: *Ramichloridiopsis wilsoniae* M. Silva & R.W. Barreto

*Etymology*: Named after morphological similarity to *Ramichloridium*.

Colonies on trichomes. External mycelium superficial, branched, septate, pale brown, smooth. Conidiophores formed apically on trichomes, in loose groups, micronematous, cylindrical, septate, occasionally branched, light brown. Conidiogenous cells holoblastic, terminal or intercallary, integrated, cylindrical, light brown, smooth. Conidiogenous loci grouped at conidiogenous cell apex, semidenticulate, slightly pigmented. Conidia holoblastic, catenulate, fusiform to cylindrical, 2-septate, hilum unthickened, not darkened, light brown, thick-walled, verrugose, with striations; schizolitic secession.

*Ramichloridiopsis wilsoniae* M. Silva & R.W. Barreto, sp. nov. Figs 14 and 15

[urn:lsid:mycobank.org:names: 810528]

*Type species*: *Ramichloridiopsis wilsoniae* M. Silva & R.W. Barreto

*Etymology*: From the host *Dimorphandra wilsonii*.

Colonies hypophyllous, effuse, brown, on trichomes. Internal mycelium absent. External mycelium superficial, 3–4 μm, branched, septate, pale brown, smooth. Conidiophores formed apically on trichomes, in loose groups, micronematous, cylindrical, 12.5–37.5 × 4–5μm, 0–2 septate, occasionally branched, light brown, smooth. Conidiogenous cells holoblastic, terminal or intercalary, integrated, cylindrical, 10.0–15× 4.5–5μm, light brown. Conidiogenous loci, 3–8 per cell, grouped at conidiogenous cell apex, semidenticulate, slightly darkened, up to 1 μm diam. Conidia dry, catenulate, fusiform to cylindrical, 15–35 × 5–7.5 μm, 2-septate, hilum unthickened, not darkened, eguttulate, light brown, thick-walled, verrugose-striate.

*In culture*: On PCA slow-growing (3.7- 4cm diam, after 27 days), circular, low convex, floccose aerial mycelium centrally with faint diurnal zonation, followed by narrow periphery of dense rosy buff immersed myceliun, ochraceous reverse; no sporulation.

*Specimens examined*: On living leaves of *Dimorphandra wilsonii*. BRAZIL: Minas Gerais: Paraopeba, Fazenda Tabuleiro Grande, 26 Jul 2011, M. Silva (VIC 31803− Holotype).

*Notes*: *Ramichloridiopsis* is phylogenetically distinct but closely related to species belonging to the *Ramichloridium* Stahel ex de Hoog and *Radulidium* Arzanlou, W. Gams & Crous (Fig 2). The genus *Ramichloridium* accommodates a wide range of species that differ in morphology and niche. Arzanlou et al. [64] used information on morphological characteristics, conidial ontogeny and DNA sequences to reallocate some species belonging to this genus and to segregate new genera. The genus *Radulidium* was segregated from *Ramichloridium* based on two species (*R*. *subulatum* and *R*. *epichloës*) distinguished by their slightly differentiated conidiophores and prominent, blunt, very dense conidium-bearing denticles [64]. *Ramichloridiopsis* is morphologically similar to *Ramichloridium* and *Radulidium*, especially in terms of its conidiogenous cells with prominent conidium-bearing denticles. However, it differs significantly from both genera by having 2-septate, catenate, thick-walled, verrugose-striate conidia.

*Stomiopeltis suttoniae* (J. M. Mend.) Luttrell, *Mycologia* 38:572 (1946) Fig 16

**Fig 16:**
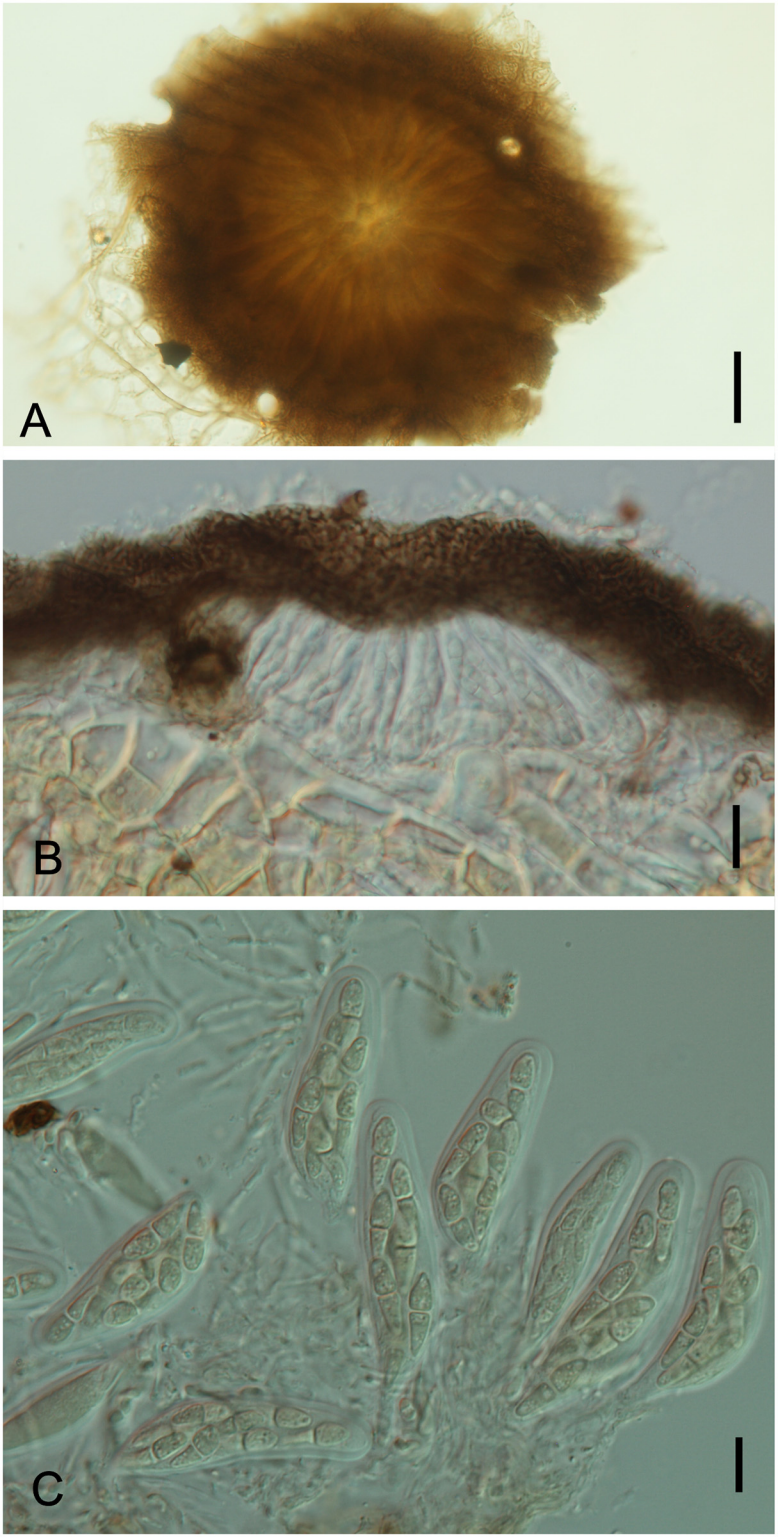
*Stomiopeltis suttoniae* on *Dimorphandra wilsonii*. (A) Upper view of shield-like ascoma. (B) Cross section of an ascoma. (C) Bitunicate asci with 1-septate hyaline ascospores. Bars: 50 μm (A); 20 μm (B); 10 μm (D).

Colonies on living leaves, hypophyllous, sparse, fuliginous, covering part of the leaf. Internal mycelium not observed. External mycelium hypogenous, anastomosing, net-forming, slightly undulate, composed of pale brown, flattened, thin-walled, septate hyphae, 2.5–5.0 μm, smooth. Ascomata hypophyllous, superficial, solitary, scutellate, ostiolate, 100–350 μm diam., composed of meandrically interwoven hyphae (textura epidermoidea), black. Pseudoparaphyses filiform, 2–2.5 μm, septate, unbranched, hyaline. Asci bitunicate, radially arranged, clavate to ovate—oblong, 35–55 × 10–15 μm, 8–spored. Ascospores obovoid to ellipsoid, 10–17.5 × 4–5.0 μm, 1–septate, upper cell slightly broader, guttulate, hyaline, smooth.

*Specimens examined*: On living leaves of *Dimorphandra wilsonii*. BRAZIL Minas Gerais: Paraopeba, Fazenda Tabuleiro Grande, 14 Jul 2009, M. Silva (VIC 31809, VIC 31810); on living leaves of *Dimorphandra wilsonii*. BRAZIL: Minas Gerais: Caetanópolis, Fazenda São Bento, 15 Jul 2009, M. Silva (VIC 31811).

*Notes*: The fungus on *D*. *wilsonii* fits well within the description of *S*. *suttoniae* (Mendonza) Luttrell [65, 66]. This species was previously reported on *Suttonia lessertiana* (A. DC.) Mez in Hawaii and on *Erythroxylum* sp. in association with *Micropeltis gravataensis* Bat. & Vital and *Hymenopeltis erythroxylii* Bat. & Vital in Brazil [65]. This is the first report of this fungus on *D*. *wilsonii* (Fabaceae). Although recorded sporadically, the few existing records of this easily overlooked species suggest that this is a broadly spread species with a wide host range.

*Trichomatomyces byrsonimae* Dornelo-Silva & Dianese, *Mycologia* 96: 879–884 (2004) Fig 17

**Fig 17:**
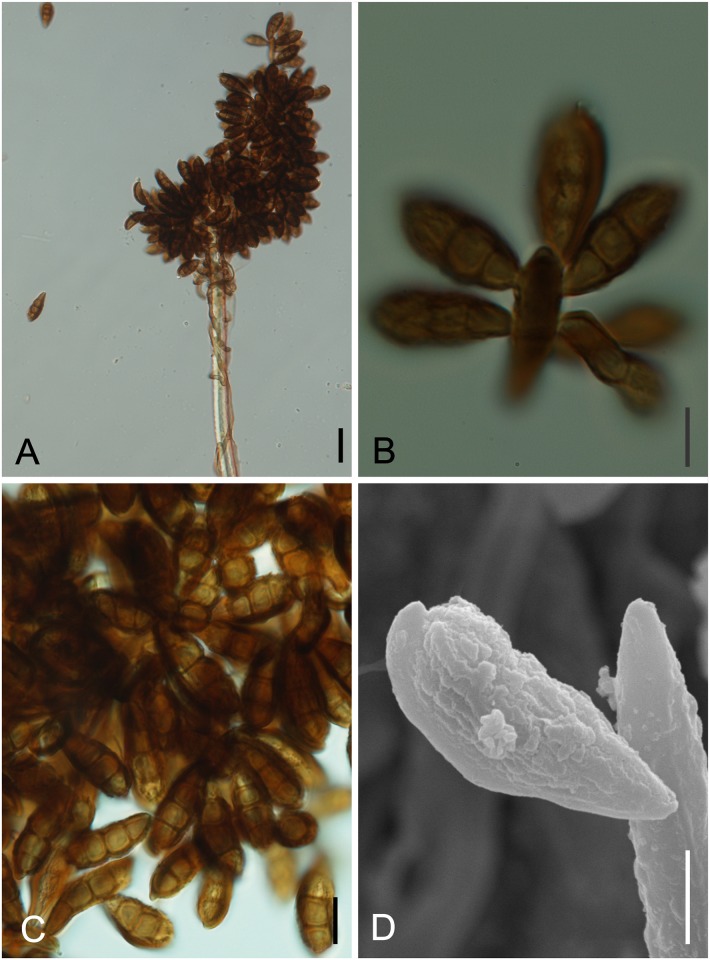
*Trichomatomyces byrsonimae* on *Dimorphandra* spp. (A) Dense, dark brown to black colony spirally arranged on trichome apex. (B) Polyblastic, condiogenous cell. (C) Conidia constricted at the septa at one side. (D) SEM of conidia. Note one side smooth and one side roughened. Bars: 20 μm (A); 10 μm (B, C); 5 μm (D).

Colonies minute, hypophyllous, dense, fuliginous, dark brown to black, forming spirally arranged heads on the apex of trichomes. Internal mycelium absent. External mycelium superficial, 2.5–5μm, branched, septate, light brown, smooth. Conidiophores crowded on apex of trichomes, single, micronematous, straight to curved, 11–21.5 × 4–5μm, 0–2 septate, unbranched, dark brown, smooth. Condiogenous cells integrated, polyblastic, sympodial, 7.5–15× 3.5–5.0 μm, 0–2 septate, brown. Conidiogenous loci indistinct. Conidia dry, solitary, elliptic-fusiform, slightly unilaterally curved, 12.5–25 × 7.5–12.5 μm, walls thinner and smooth on one side and thicker and roughened on the opposite side, apex acute and base truncate, 1–4 septate, constricted at septae on one (thinner-walled) side but not on the other (thicker-walled) side, brown, eguttulate, striate.

*Specimens examined*: On living leaves of *Dimorphandra wilsonii*. BRAZIL: Minas Gerais: Paraopeba, Fazenda Tabuleiro Grande, 13 Jul 2009, M. Silva (VIC 31769); on living leaves of *Dimorphandra mollis*. BRAZIL: Minas Gerais: Paraopeba, Floresta Nacional de Paraopeba, 15 Jul 2009, M. Silva (VIC 31777); on living leaves of *Dimorphandra wilsonii*. BRAZIL: Minas Gerais: Caetanópolis, Fazenda São Bento, 19 Jul 2010, M. Silva (VIC 31778); on living leaves of *Dimorphandra wilsonii*. BRAZIL: Minas Gerais: Paraopeba, Fazenda Tabuleiro Grande, 26 Jul 2011, M. Silva (VIC 31799); 26 Jul 2011, M. Silva (VIC 31800); 26 Jul 2011, M. Silva (VIC 31801).

*Notes*: *Trichomatomyces byrsonimae* (Bat. & Peres) Dornelo-Silva & Dianese is recorded here for the first time on *D*. *wilsonii* and *D*. *mollis*. This species was originally described as *Piricauda byrsonimae* Bat. & Peres [37] on *Byrsonima basiloba* A. Juss (Malpighiaceae) and later transferred to a new genus (*Trichomatomyces*) by Dornelo-Silva and Dianese [67]. The fungus collected on *Dimorphandra* spp. fitted well within the description of *T*. *byrsonimae* given in Dornelo-Silva and Dianese [67]. The material examined by the latter authors was growing on trichomes of *Qualea grandiflora* Mart. (Vochysiaceae). Although poorly known and only recorded three times, this finding indicates that this fungus is not host-specific because each record was from a host belonging to a distinct family. All records are from the Cerrado.

*Vesiculohyphomyces cerradensis* Armando, Pereira-Carvalho & Dianese, Mycol. Res. 113: 261–274 (2009) Figs 18 and 19

**Fig 18:**
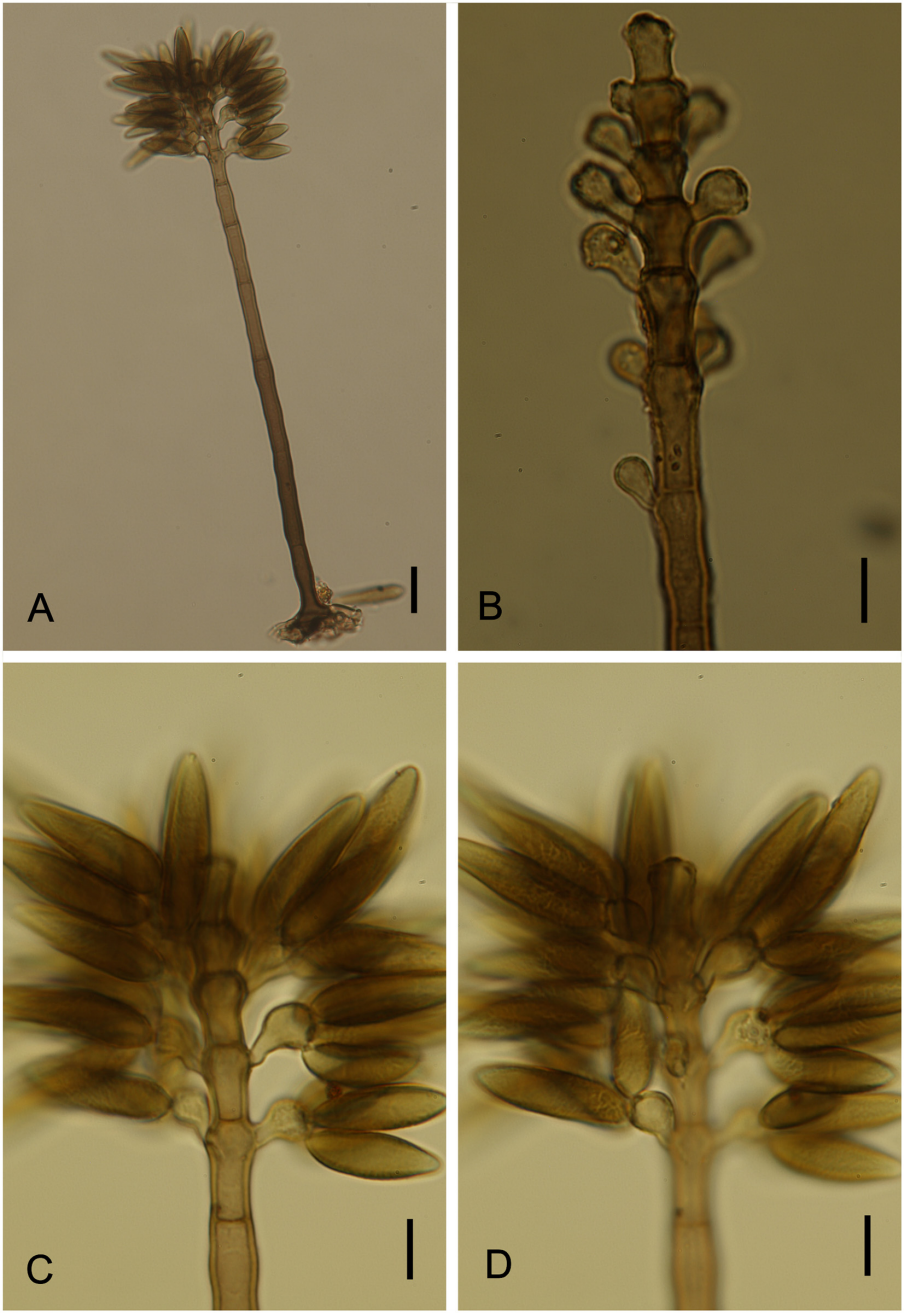
*Vesiculohyphomyces cerradensis* on *Dimorphandra wilsonii*. (A) Macronematous, cylindrical, solitary conidiophore. (B) Whorls of polyblastic, vesicle-shaped, conidiogenous cells at apex of conidiophore. (C) Fertile conidiophores bearing numerous fusiform, conidia. (D) *Ibid* at different plane of focus showing wall sculpturing. Bars: 20 μm (A); 10 μm (B, C, D).

**Fig 19:**
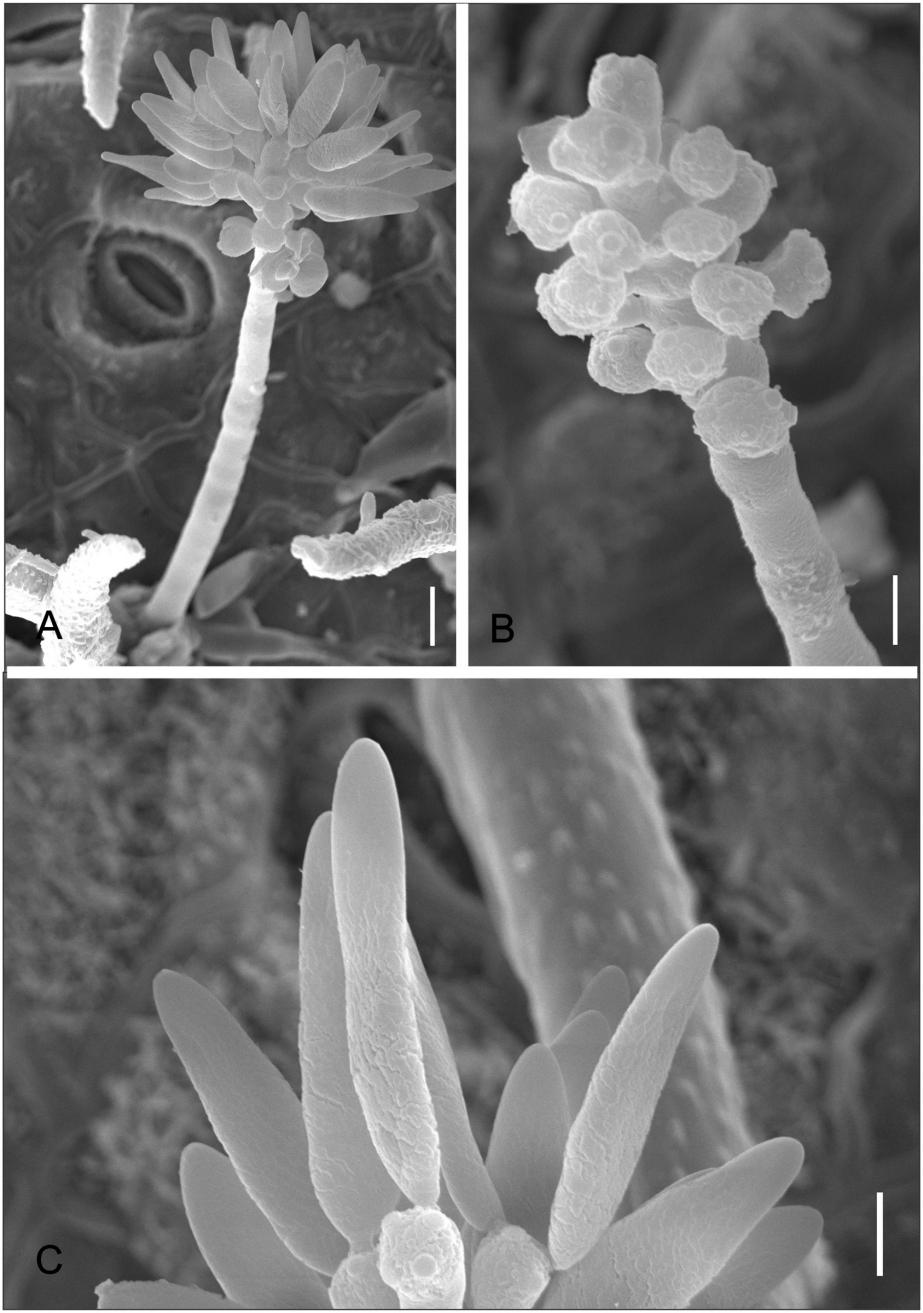
SEM of *Vesiculohyphomyces cerradensis* on *Dimorphandra wilsonii*. (A) Macronematous conidiophore. (B) Close-up of conidiogenous cells at upper, fertile part of conidiophore. (C) Close-up of faintly striate conidia. Bars: 10 μm (A); 5 μm (B, C).

Colonies hypophyllous, brown, sooty. Internal mycelium absent. External mycelium superficial, 4–5 μm, branched, septate, brown, smooth. Conidiophores hypophyllous, solitary, macronematous, cylindrical, 255–362.5 × 5–7.5 μm, 9–16 septate, unbranched, dark brown, smooth. Conidiogenous cells in terminal clusters of verticillate rings on conidiophores, discrete, polyblastic, each representing an inflated subsphaerical vesicle-like head on a short and somewhat crooked stalk, 5–12.5 × 4–7.5 μm, light brown. Conidiogenous loci 2–5 per cell, 1.5–2 μm diam, non-cicatrized. Conidia dry, in groups on conidiogenous cell, fusiform to cymbiform, 12.5–39.5 × 5–7.5 μm, conidial apex acute, 2–2.5 μm, base rounded, 4–5 μm, 1–4 euseptate, hilum slightly protuberant, eguttulate, striate when mature.

*Specimens examined*: On living leaves of *Dimorphandra wilsonii*. BRAZIL: Minas Gerais: Paraopeba, Fazenda Tabuleiro Grande, 26 Jul 2011, M. Silva (VIC 31802).

*Notes*: The monotypic genus *Vesiculohyphomyces* is characterized by the presence of discrete vesicle-shaped conidiogenous cells arranged in apical verticillate rings on conidiophores and conidia that are solitary, pigmented and striate when mature. The fungus collected on *D*. *wilsonii* fits well within the description of *V*. *cerradensis*. This species was previously known only from trichomes of *Caryocar brasiliense* Cambess. (Caryocaraceae) from the Brazilian Cerrado [68]. The occurrence of this fungus on two unrelated hosts suggests that it is a generalist epiphyte on Cerrado plants. *Vesiculohyphomyces cerradensis* is recorded here for the first time colonizing *D*. *wilsonii*.

## Conclusions

A high level of fungal diversity was found during the exploration of the foliicolous mycobiota of *D*. *wilsonii* (fourteen species). This diversity is significantly higher than the estimated average of six fungal species expected for each plant species roughly suggested by [69]. Additionally, this list is clearly only partial because a whole range of fungi occupying other niches in the plant remained unexplored in this work. Interestingly, *D*. *wilsonii* and *D*. *mollis* shared few fungi in our study despite their taxonomic relatedness (*Peudocercospora dimorphandraea*, *Pseudocercosporella dimorphandraea*, *P*. *paraguayensis* and *T*. *byrsonimae*). Six fungal taxa were found on *D*. *mollis*, including one that was described in a separate publication as the new genus *Alveariospora* Meir. Silva, R.F. Castañeda, O.L. Pereira & R.W. Barreto [70] and *Johansonia chapadensis* [47], which had its host tentatively identified in the original publication as *D*. *mollis* (confirmed here after being recollected on *D*. *mollis*). Several of the fungi were recognized as polyphagous/generalist organisms of little relevance in terms of conservation (*T*. *byrsonimae*, *P*. *atra*, *M*. *dipterygis* and *S*. *suttoniae*). Interestingly, several of these fungi appeared to be particularly rare in our sampling. Several were limited to very few leaves (*V*. *cerradensis*, *P*. *paraguayensis*, and *G*. *polystigmatis*), whereas others were either abundant on each specimen, frequently collected or both (*T*. *byrsonimae*, *P*. *atra*, *M*. *dipterygis* and *S*. *suttoniae*). The discovery of three new fungal taxa seemingly restricted to *Dimorphandra wilsonii*, including one that was described as belonging to a new genus (*Janetia wilsoniae*, *Byssogene wilsoniae*, and *Ramichloridiopsis wilsoniae*) mirrors the results of the pioneering work on the mycobiota of the endangered Brazilian plant species *C*. *floccosa* by Rocha et al. [8]. In that work, six novel fungal taxa were discovered on this host, including a new genus. This may be the best indication that the loss of a single plant species such as *D*. *wilsonii* can have disastrous consequences for a unique portion of the mycosphere. Additional studies are needed to confirm this conjecture and to demonstrate that the taxa found only on *D*. *wilsonii* are strictly host-specific and not capable of surviving on other substrates (i.e., as saprophytes or endophytes on other hosts); moreover, studies should be conducted to test the hypothesis of the risk of co-extinction. Fortunately, the effort coordinated by F. M. Fernandes to survey and preserve existing individuals occurring in the wild and to reintroduce *D*. *wilsonii* in areas where it originally existed have produced good results. The original list of 12 remaining individuals of *D*. *wilsonii* was significantly raised by the continuation of the searches for the remaining plants (now the count is of 219 mature individuals occurring in nature) and area of distribution is larger than previously though (now 16 municipalities in the state of Minas Gerais are known to harbor this endangered plant). Additionally the plant is being progressively re-introduced in areas where it occurred in the past and a National Action Plan for its protection has been recently published [12]. There is hope that the actions included in the plan will eventually result in the long-term preservation of this tree species and as a consequence the preservation of its specialized mycobiota.
